# Mutation in pore-helix modulates interplay between filter gate and Ba^2+^ block in a Kcv channel pore

**DOI:** 10.1085/jgp.202313514

**Published:** 2024-04-23

**Authors:** Noel Tewes, Beatrice Kubitzki, Flandrit Bytyqi, Nikola Metko, Sebastian Mach, Gerhard Thiel, Oliver Rauh

**Affiliations:** 1 Membrane Biophysics, Technische Universität Darmstadt, Darmstadt, Germany; 2 Centre for Synthetic Biology, Technische Universität Darmstadt, Darmstadt, Germany

## Abstract

The selectivity filter of K^+^ channels catalyzes a rapid and highly selective transport of K^+^ while serving as a gate. To understand the control of this filter gate, we use the pore-only K^+^ channel Kcv_NTS_ in which gating is exclusively determined by the activity of the filter gate. It has been previously shown that a mutation at the C-terminus of the pore-helix (S42T) increases K^+^ permeability and introduces distinct voltage-dependent and K^+^-sensitive channel closures at depolarizing voltages. Here, we report that the latter are not generated by intrinsic conformational changes of the filter gate but by a voltage-dependent block caused by nanomolar trace contaminations of Ba^2+^ in the KCl solution. Channel closures can be alleviated by extreme positive voltages and they can be completely abolished by the high-affinity Ba^2+^ chelator 18C6TA. By contrast, the same channel closures can be augmented by adding Ba^2+^ at submicromolar concentrations to the cytosolic buffer. These data suggest that a conservative exchange of Ser for Thr in a crucial position of the filter gate increases the affinity of the filter for Ba^2+^ by >200-fold at positive voltages. While Ba^2+^ ions apparently remain only for a short time in the filter-binding sites of the WT channel before passing the pore, they remain much longer in the mutant channel. Our findings suggest that the dwell times of permeating and blocking ions in the filter-binding sites are tightly controlled by interactions between the pore-helix and the selectivity filter.

## Teaser

Tewes et al. use a simple K^+^ channel pore to examine filter gating. Voltage-dependent closings in pore-helix mutants can be explained by an elevated sensitivity to block by trace contaminations of Ba^2+^. This means that the dwell time of ions in the filter determines the closed times of the filter gate.

## Introduction

Opening and closing of K^+^ channels is in most cases determined by two types of gates: the so-called inner gate is formed by dynamic hydrophobic barriers that control entry into and exit from the cavity on the cytosolic side (Yellen, 2002; Kim and Nimigean, 2016; Kopec et al., 2019). The filter gate faces the extracellular side of the channel protein and is part of the selectivity filter structure where it controls the current through the filter (Cuello et al., 2010; Kim and Nimigean, 2016; Kopec et al., 2019). After discovering filter gating in KcsA (Cordero-Morales et al. 2006, 2007), structural and functional studies have implied that this type of gating is a general phenomenon in K^+^ channels including physiologically important Kir (Sackin et al., 2009) and Kv channels (Köpfer et al., 2012) as well as K2P channels (Natale et al., 2021).

Gating in the filter is generated and modulated by critical interactions between the selectivity filter and surrounding parts of the protein (Cordero-Morales et al., 2007; Cuello et al., 2010; Kim and Nimigean, 2016; Kopec et al. 2019, 2023). This mechanical interplay between the filter and supporting scaffold causes small positional changes in the delicate filter geometry, which then favor or disfavor current through the filter. A prominent example of this kind of filter gating is C-type inactivation. It occurs for instance in several Kv channels after voltage-dependent opening of the inner gate (Peters et al., 2013). Also, in K2P channels, the selectivity filter interacts with the surrounding protein (Niemeyer et al., 2016; Piechotta et al., 2011; Lolicato et al., 2020) and translates in this manner the effect of external regulatory inputs into a modulation of filter gating kinetics in these channels.

In a recent study, we used mutagenesis and single-channel recordings of the small channel Kcv_NTS_ to obtain more general insight into structure/function correlates of filter gating (Rauh et al., 2022). This channel is particularly suitable for addressing this question because it exhibits distinct voltage-dependent closures at negative voltages, which can be assigned to filter gating (Rauh et al., 2018). Also, since the Kcv_NTS_ channel has no inner gate (Rauh et al., 2017), these fast open–closed transitions at negative voltages must be entirely generated by operation of the filter gate.

**Figure 1.**
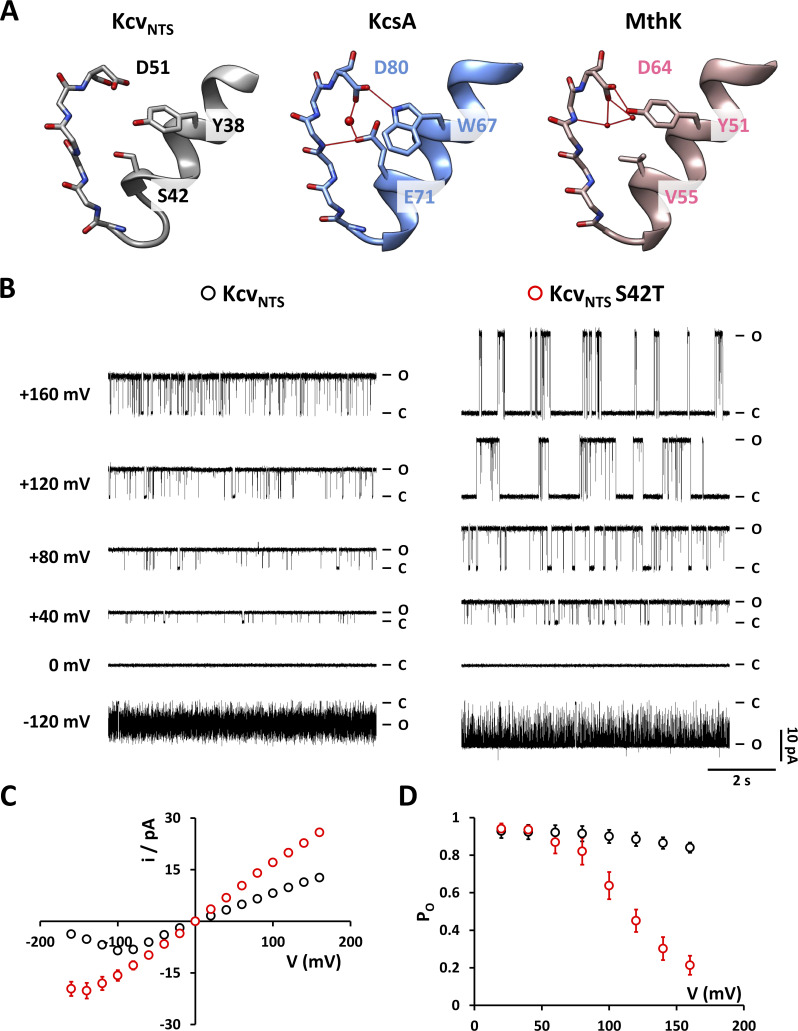
**Substitution of the critical amino acid S42 at the C-terminal end of the pore-helix by threonine has dramatic impacts on gating and K**^**+**^ **conductance of Kcv**_**NTS**_**. (A)** Selectivity filter and pore helix of Kcv_NTS_ (grey, homology model on KirBac1.1 [PDB ID 1P7B] calculated with Swiss model), KcsA (blue, PDB ID 1K4C), and MthK (pink, PDB ID 3LDC). Some of the hydrogen bonds are drawn in red for KcsA and MthK (as identified by UCSF chimera) with the red spheres being buried water molecules. **(B)** Representative single-channel current traces recorded in DPhPC bilayers at constant voltages between −160 and +160 mV from Kcv_NTS_ and Kcv_NTS_ S42T. The closed and open levels are marked by C and O, respectively. **(C and D)** Current–voltage relationships and open probabilities (P_O_) from time series like those in B. Symbols as in B. Data points show arithmetic mean ± SD (often hidden under the data points) of at least three independent measurements.

**Figure S1.**
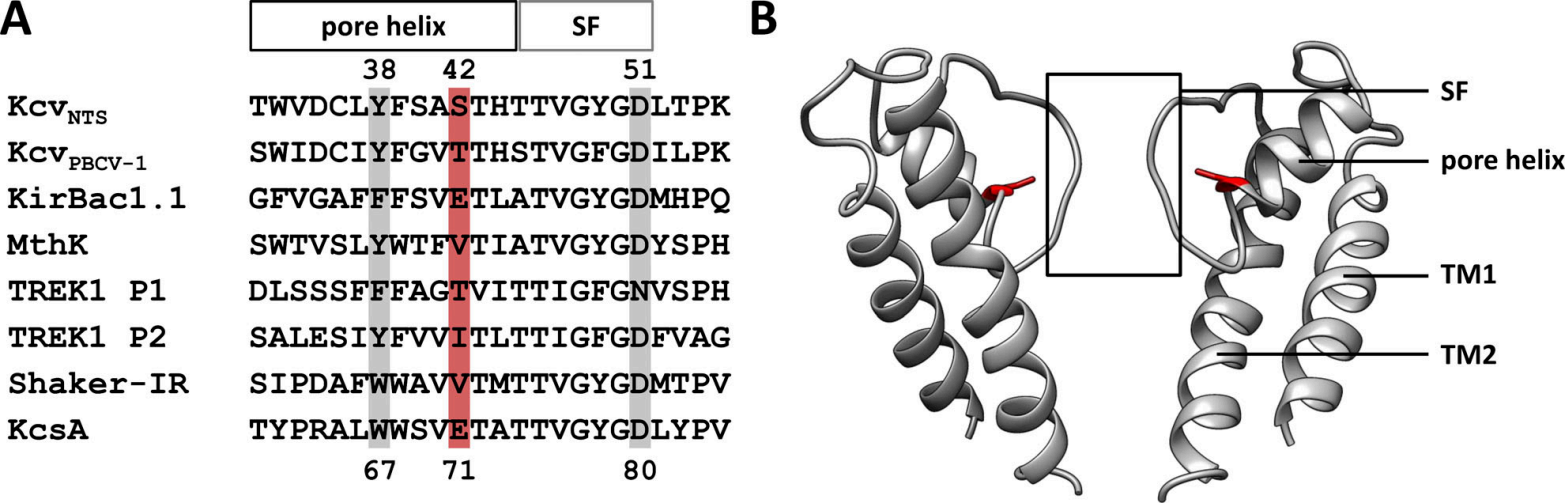
**Crucial amino acids for filter gating in Kcv**_**NTS**_ **and other K**^**+**^ **channels. (A)** Multiple sequence alignment for the pore-loop consisting of the pore helix and the selectivity filter (SF) for different K^+^ channels. The locations of the three most important residues anchoring the selectivity filter to the pore helix are highlighted. Residue numbers for Kcv_NTS_ and KcsA are given on the top and bottom, respectively. **(B)** Side view of two opposing subunits of Kcv_NTS_.The homology model was calculated with the Swiss model (Waterhouse et al, 2018) using KirBac1.1 (PDB ID 1P7B; Kuo et al, 2003) as a template. The amino acid S42 is highlighted in red.

It was already shown that the position S42 in Kcv_NTS_ is structurally equivalent to the amino acid E71 in KcsA and V55 in MthK (Fig. 1 A and Fig. S1 A), a position which is critical for filter gating in the latter channels (Rauh et al., 2022; Kopec et al., 2023; Chakrapani et al., 2011). As in the case of KcsA and MthK, mutations of this critical position in the pore-helix of Kcv_NTS_ also affect filter gating. It was shown that mutations S42T and S42V slow down the closing of the filter gate in the latter channel (Rauh et al., 2022). In addition to its effect on fast gating, these two mutations but also the S42A mutation elicited an additional gating phenomenon: all three mutants introduce long-lasting and voltage-dependent closures at positive voltages. The data suggested at first glance that mutations at the critical position 42 modulate in addition to the aforementioned fast gating, a second gate. In the absence of an inner gate in this channel, the data suggest that voltage-dependent closure at positive voltages is the result of an additional filter gating mode. Here, we show that this additional voltage-dependent closing at positive voltages is not generated by an additional gating mode of the filter gate but by an increase in the sensitivity of the selectivity filter to a voltage-dependent block by another ion. Our data show that the S42T mutation generates a 200-fold increase in the sensitivity of the channel to cytosolic Ba^2+^. Because the binding site for the Ba^2+^ block is in these channels, part of the selectivity filter we predict that manipulations of the coupling between pore-helix and selectivity filter are not only affecting the process of gating but also the fine structure of the selectivity filter, which determines the propensity of an entering ion to permeate or block the channel pore.

## Materials and methods

### Cloning, mutagenesis, protein expression, and purification

Serine in position 42 in the Kcv_NTS_ channel was mutated into all other 19 proteinogenic amino acids by site-directed mutagenesis using a protocol based on the method described in (Braman et al, 1996). The coding regions of all constructs were sequenced. In vitro protein expression and purification of the Kcv_NTS_ channel and its mutants were performed as described previously (Rauh et al., 2022) in an in vitro reaction with the Expressway Mini Cell-Free Expression System (Invitrogen). The nascent channel proteins were thereby directly embedded into lipid nanodiscs. The latter MSP1D1-His DMPC nanodiscs (Cube Biotech GmbH) contain multiple His-tags, which served in the next step for purification by metal chelate affinity chromatography (0.2 ml HisPur Ni-NTA spin columns, Thermo Fisher Scientific). Different from the manufacturer’s instructions, the washing and elution buffers were salt-free. The column was washed three times with 400 μl of 20 mM imidazole, and the nanodisc–channel complexes were eluted in three fractions (200 μl each) with 250 mM imidazole. This improves the reconstitution efficiency into the bilayer. For details of the procedure see Rauh et al. (2021).

### Functional reconstitution of channel proteins in planar lipid bilayers

Functional reconstitution of channel proteins in planar lipid bilayers was performed as in previous studies (Rauh et al. 2017, 2021). Cis and trans chambers were filled with standard recording buffer (100 mM KCl [AppliChem GmbH], 10 mM HEPES [AppliChem GmbH] in double-distilled water, and adjusted to pH 7.4 with KOH). Bilayers were formed from 1,2-diphytanoyl-sn-glycero-3-phosphocholine (DPhPC, Avanti Polar Lipids) by the pseudo painting/air bubble technique. For single-channel recordings, an elution fraction was diluted in 250 mM imidazole solution 1:1,000–1:100,000 and 1–3 μl was added directly below the bilayer in the trans compartment with a bent Hamilton syringe. Insertion and orientation of a single-channel protein were monitored during short-voltage steps. The pronounced asymmetry of Kcv_NTS_ channel conductance allowed the determination of channel orientation in the bilayer. The trans compartment was grounded, and membrane voltages were applied to the cis compartment. With a strong bias of the Kcv_NTS_ protein for inserting exclusively with the cytoplasmic side first into the bilayer, positive currents correspond to an outward current in the in vivo situation.

Steady-state single-channel currents were recorded at room temperature (∼25°C) over a voltage window from +160 to −160 mV in steps of 20 mV for 1–5 min. Both chambers were connected via Ag/AgCl electrodes to an amplifier (L/M-EPC-7; List-Medical). Currents were filtered with a 1-kHz four-pole Bessel filter before digitization with a sampling frequency of 5 kHz (LIH 1600; HEKA Elektronik).

For block experiments with divalent cations, an appropriate amount of a stock solution containing 10 mM of the divalent ion with Cl^−^ as anion was added to the cis (= intracellular) or trans (= extracellular) chamber and carefully mixed by repeated pipetting. Contaminations of Ba^2+^ in the KCl buffer were eliminated according to Neyton (1996) by adding 200 µM of the chelating macrocyclic polyether (+)-18-Crown-6-tetracarboxylic acid (18C6TA; Aldrich Chem. Co.) to the buffer solution.

### Data analysis and statistics

The voltage dependency of the open probability P_O_ of Kcv_NTS_ and its mutants was quantified by fitting P_O_/V data with the Boltzmann equation:

$$P_{O}=\frac{P_{O,\max}-P_{O,\min}}{1+\exp\left[\frac{\delta zF}{RT}\left(V_{1/2}-V\right)\right]}+P_{O,\min}\text{,}$$ (1)

where P_O,max_ is the maximal and P_O,min_ the minimal open probability, δz is the apparent gating valence, F is the Faraday constant, R the gas constant, T is the absolute temperature during the experiment in Kelvin (T = 298 K), V_1/2_ is the voltage at half-maximal P_O_, and V is the membrane voltage. In most cases, P_O,min_ was set to zero.

To examine single-channel gating kinetics, the open and closed lifetimes obtained by a higher-order Hinkley detector (H.O.H.D.) (Schultze and Draber, 1993) were used for dwell-time analyses. The H.O.H.D. is an extension of the simple Hinkley detector, an algorithm based on the recursive calculation of the cumulative sum g_t_ for all time points t (t = 0,1,2,...N) of a time series z_t_:

$$g_{t}=g_{t-1}+\left(z_{t}-\frac{{µ}_{0}+{µ}_{1}}{2}\right)\text{,}$$ (2)

with g_t−1_ being the value of the test function g at time t − 1, z_t_ the value of the analyzed time series at time t, and µ_0_ and µ_1_ (µ_1_ > µ_0_) being the current values of the closed and open levels, respectively. If the calculated value for g_t_ is negative, it is immediately set to 0:

$$g_{t}=\left\{ \begin{array}{l} 0\;if\;g_{t}<0\\ g_{t\;}if\;g_{t}\geq 0 \end{array}\right.\text{.}$$ (3)

Consequently, g_t_ remains at 0 as long as z_t_ does not exceed the value (µ_0_ + µ_1_)/2. If z_t_ jumps from µ_0_ to µ_1_, g_t_ increases. A jump is detected if the test value g_t_ exceeds a threshold value λ. The threshold value was calculated as follows:

$$\lambda =\frac{22p}{SNR^{2}}\text{,}$$ (4)

with p being the half-open channel amplitude and SNR the signal-to-noise ratio. For details, see Schultze and Draber (1993). The H.O.H.D. does not use the simple but the eighth-order cumulative sum of the test value g_t_, which is calculated as follows:

$$g_{t}^{1}=g_{t-1}^{1}+\left(z_{t}-\frac{{µ}_{0}+{µ}_{1}}{2}\right)$$

$$g_{t}^{2}=g_{t-1}^{2}+g_{t}^{1}$$

$$g_{t}^{3}=g_{t-1}^{3}+g_{t}^{2}$$

⋮

$$g_{t}^{8}=g_{t-1}^{8}+g_{t}^{7}\text{.}$$ (5)

The use of g_t_^8^ instead of the simple cumulative sum g_t_ improves the attenuation of high-frequency noise.

A jump is detected as soon as the test value g_t_^8^ exceeds the threshold value λ^8^. The incidence of the jump is estimated as the time at which g_t_ last adopted the value 0. Subsequently, g_t_ is set back to 0 and the algorithm starts again with the opposite sign, as it now searches for a jump from µ_1_ to µ_0_.

The theoretical temporal resolution limit t_res_ of the H.O.H.D. (as an integer multiple of the sampling interval) was

$$t_{res}=\frac{22}{SNR^{2}}\text{.}$$ (6)

For dwell time analysis, only measurements at membrane voltages greater than or equal to +60 mV were used. The lowest SNR value was achieved at +60 mV and was always at least SNR = 20. Consequently, the effective temporal resolution limit of our analysis was not determined by the H.O.H.D. but by the 1-kHz four-pole Bessel filter used in our measurement setup. The latter has a rise time T_r_ of about 400 µs. We therefore assumed that virtually all events with a duration ≥1 ms were detected correctly and that the majority of missed events and events registered incorrectly in terms of their duration fall within the interval t = [0, 1 ms]. For this reason, we only considered events with a duration of >1 ms for the dwell-time analysis.

The dwell-time histograms were then generated by grouping the lifetimes of open and closed events into bins with exponentially growing widths. To obtain the mean lifetimes of open- and closed-time populations, a multiexponential probability density function (*pdf*) was fitted to the dwell-time histograms:

$$pdf\left(t\right)=\sum_{j=1}^{n}\frac{a_{j}}{\tau_{j}}e^{-\frac{t}{\tau_{j}}}\text{,}$$ (7)

with τ_j_ being the mean lifetime, a_j_ the area of the jth component, and n the number of exponential components. For details, see Colquhoun and Hawkes (1995). All open-time histograms could be fitted with a single-exponential *pdf*, while closed-time histograms required up to four exponential components.

To be able to assume that the mean lifetimes of the closed-time populations correspond to the true mean lifetimes, only those measurements were used whose mean open time was >20 ms. In this way, the number of open events with t < 1 ms should be <5% and therefore negligible. A missed events correction of the mean closed times was therefore not carried out.

Inspection of the closed-time histograms, on the other hand, implies that a large number of short closed events (<1 ms) were not detected and the mean open time was thus overestimated. Consequently, the mean open time determined by fitting Eq. 7 to the open time histograms had to be corrected. This was done as follows: the constructed closed-time histograms were fitted with Eq. 7 in the time interval [1 ms, ∞]. For the number of closed events N_obs_ observed in this interval, the following must apply:

$$N_{obs}=N_{total}\int_{t_{d}}^{\infty }pdf\left(t\right)dt=N_{total}\sum_{j=1}^{n}a_{j}e^{-\frac{t_{d}}{\tau_{j}}}\text{,}$$ (8)

with N_total_ being the total number of theoretically expected closed events and t_d_ being the time resolution limit set to 1 ms. Since the total number of closed events N_total_ is the sum of the number of observed events N_obs_ and the number of missed events N_missed_, the number of missed closed events could be calculated as follows:

$$N_{missed}=N_{obs}\left[{\left(\sum_{j=1}^{n}a_{j}e^{-\frac{t_{d}}{\tau_{j}}}\right)}^{-1}-1\right]\text{.}$$ (9)

In order to obtain an approximation of the true mean open lifetime τ_O_, we assumed (1) that the missed closed events are randomly distributed over the open events and (2) that the missed closed events have a negligible effect on the total time of the channel in the open state:

$$\tau_{O}^{obs}N_{obs}\approx \tau_{O}N_{total}=\tau_{O}\left(N_{obs}+N_{missed}\right)\text{.}$$ (10)

The true mean open time *τ*_*O*_ was therefore estimated from the observed mean open time $\tau_{O}^{obs}$ and the calculated number of missed closed events N_missed_ as follows:

$$\tau_{O}\approx \tau_{O}^{obs}\frac{N_{obs}}{\left(N_{obs}+N_{missed}\right)}\text{.}$$ (11)

**Scheme 1:**
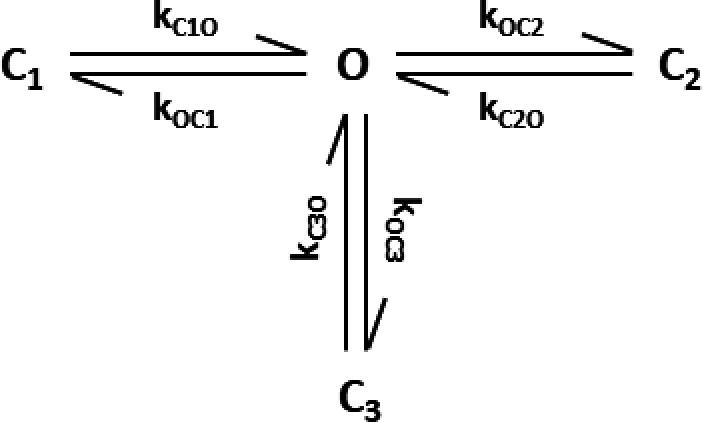


Each closed-time population was then treated as an individual closed-state Cj that can be reached exclusively from the open state O as shown in Scheme 1.

This assumption allowed the calculation of the occupation probabilities P_Cj_ and the apparent rate constants k_OCj_ as follows:

$$P_{Cj}=\frac{a_{Cj}\tau_{Cj}}{\tau_{O}+\sum_{j=1}^{n}a_{Cj}\tau_{Cj}}\text{,}$$ (12)

$$k_{OCj}=\frac{a_{Cj}}{\tau_{O}}\text{.}$$ (13)

The assumption that there are no transitions between the closed states is a simplification resulting from the lack of information about possible hidden C–C transitions. If additional information on the state transitions becomes available in the future, which requires a modification of this model topology, algorithms are available to transform equivalent models into each other (Kienker, 1989).

The renormalized amplitudes a_f_ (fast component) and a_s_ (slow component) of Ba^2+^-induced block-time populations C_2Ba_ and C_3Ba_ were calculated as follows:

$$a_{f}=\frac{1}{\tau_{C2Ba}}\frac{a_{C2Ba}\tau_{C2Ba}}{a_{C2Ba}\tau_{C2Ba}+a_{C3Ba}\tau_{C3Ba}}\text{,}$$ (14a)

$$a_{s}=\frac{1}{\tau_{C3Ba}}\frac{a_{C3Ba}\tau_{C3Ba}}{a_{C2Ba}\tau_{C2Ba}+a_{C3Ba}\tau_{C3Ba}}\text{.}$$ (14b)

Experimental data are generally presented as arithmetic/geometric mean ± arithmetic/geometric standard deviation (SD) of N independent experiments.

The analysis of single-channel traces was performed using the program Kielpatch (https://www.zbm.uni-kiel.de/aghansen/software.html). Matlab 2014b (MathWorks, Inc.) was used for dwell-time analyses. The Matlab files are available from the corresponding author upon reasonable request by email.

### ICP-MS measurements

Metal ion contaminations in measuring buffer were analyzed using inductively coupled plasma mass spectrometry (ICP-MS, Analytik Jena Plasma Quant MS Elite). Sample preparation (double-distilled water or 100 mM KCl, 10 mM HEPES/KOH, pH 7) included filtration with a 0.45-µm regenerated cellulose (RC) filter (Perfect Flow syringe filter, Wicom) and acidification with ultrapure HNO3 (ROTIPURAN ≥69 %, Carl Roth) to pH < 2. Calibration standards in a concentration range of 0.2–100 µg/l for barium were prepared from a multielement standard (Analytik Jena). A 10-µg/l internal standard solution of Sc, Y, In, and Bi (diluted 100 mg/l Analytik Jena Internal standard solution) was added online via a peristaltic pump to all samples and standards to compensate for drift of the ICP-MS system.

### Online supplemental material

**Figure S2.**
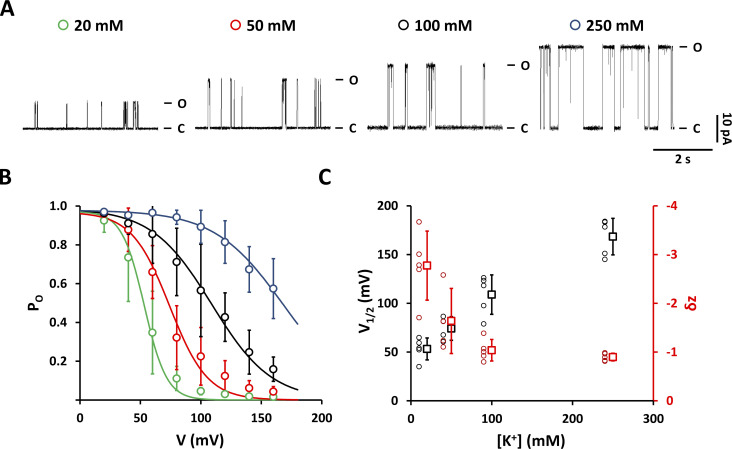
**Raising the K**^**+**^ **concentration suppresses voltage-dependent inactivation at positive voltages. (A)** Representative Kcv_NTS_ S42T single-channel current traces recorded at +160 mV in the presence of different symmetrical KCl concentrations as indicated above each trace. Closed and open levels are marked by C and O, respectively. **(B)** Open probabilities from time series like those in A. Data points correspond to symbols in A. P_O_/V relationships are well described by a single Boltzmann equation (solid lines). **(C)** Mean V_1/2_ values (open black squares) and mean gating valences δz (open red squares) were obtained by fitting P_O_/V curves as shown in B plotted against the corresponding K^+^ concentration. Individual data points are shown to the left of the mean values. Data points in B and C show arithmetic mean ± SD of at least three independent measurements.

**Figure S3.**
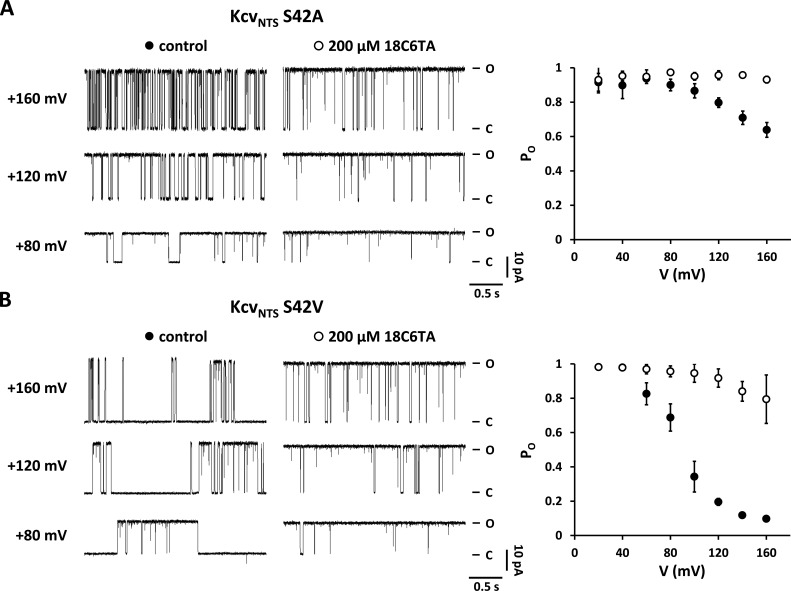
**(+)-18-Crown-6-tetracarboxylic acid (18C6TA) in the cytosolic solution abolishes voltage-dependent closures in Kcv**_**NTS**_ **S42A and Kcv**_**NTS**_ **S42V. (A and B)** Effect of 18C6TA on single-channel fluctuations of Kcv_NTS_ S42A (A) and Kcv_NTS_ S42V (B). Left: Representative single-channel current traces for both mutants at indicated membrane potentials in absence (control) and presence of 200 µM 18C6TA in the cytosolic solution. Right: Corresponding open probabilities of mutants in absence (closed circles) and presence (open circles) of 200 µM 18C6TA in the cytosolic solution. Closed and open levels in A and B are marked by C and O, respectively. Right: Open probabilities of Kcv_NTS_ S42V in the absence (closed circles) and presence (open circles) of 200 µM 18C6TA in the cytosolic solution. Data points show arithmetic mean ± SD of three independent measurements.

Fig. S1 shows crucial amino acids for filter gating in Kcv_NTS_ and other K^+^ channels. Fig. S2 shows the effect of different symmetrical K^+^ concentrations on the voltage-dependent inactivation of Kcv_NTS_ S42T. Fig. S3 shows that the addition of 18C6TA to the cytosolic solution abolishes the voltage-dependent closures in Kcv_NTS_ S42A and Kcv_NTS_ S42V.

## Results

### The conservative mutation S42T has a dramatic effect on single-channel properties of Kcv_NTS_

Single-channel activity of wild type Kcv_NTS_ and its S42T mutant (Kcv_NTS_ S42T) was recorded like in previous studies using a combination of in vitro translation into nanodiscs and functional reconstitution into DPhPC bilayers. In symmetrical 100 mM KCl, Kcv_NTS_ has a high open probability over the entire window of test voltages between −160 and +160 mV (Fig. 1, B and D). Typically, the channel shows fully resolvable open and closed events at positive voltages and very fast open/closed transitions at negative voltages (Fig. 1 B). The latter is the result of sub-millisecond gating, which can be causally linked to filter gating in this channel (Rauh et al., 2018). Functional reconstitution of Kcv_NTS_ S42T reveals channel activity with a twofold increase in unitary conductance (Fig. 1 C) as well as distinct impacts on gating (Fig. 1 B). In a previous study, we have already shown that the S42T mutation stabilizes the filter gate, resulting in a slow-down and full resolution of open/closed transitions at negative voltages (Rauh et al., 2022).

At positive voltages, Kcv_NTS_ S42T generates distinct long closures, which increase in frequency with depolarizing voltages (Fig. 1 B). At first glance, this channel closing at positive voltages has similarities to C-type inactivation in that the voltage dependency of channel inactivation is shifted toward more positive voltages with increasing KCl concentrations (Fig. S2). A plot of the P_O_ values as a function of voltage can be fitted for low and medium KCl concentrations with a single Boltzmann function (Fig. S2 B). From the fit parameters, it is evident that increasing KCl concentrations shifts the voltage for half-maximal inactivation (V_1/2_) positive and decreases the value of the apparent gating valence δz (Fig. S2 C).

### The voltage-dependent closures are kinetically reminiscent of block events

**Figure 2.**
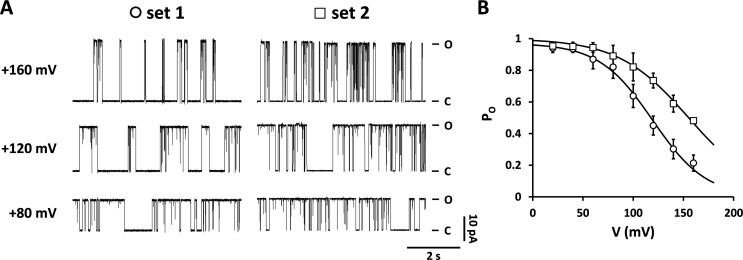
**Characteristics of voltage-dependent closure of Kcv**_**NTS**_ **S42T show large variability between different sets of experiments. (A)** Representative Kcv_NTS_ S42T single-channel current traces recorded in DPhPC bilayers from two independent sets of experiments performed over several months. Applied voltages are depicted on the left. Closed and open levels are marked by C and O, respectively. **(B)** Open probabilities from time series like those in A. Data points correspond to symbols in A and show arithmetic mean ± SD of at least three independent measurements. P_O_/V relationships are well described by a single Boltzmann equation (solid lines, Eq. 1) with V_1/2_ of 119 ± 7 and 155 ± 3 mV for experiments 1 and 2, respectively; the corresponding gating valences (δz) are −0.97 ± 0.04 e_0_ and −0.73 ± 0.16 e_0_ for experiments 1 and 2, respectively.

In a systematic study of voltage-dependent closure in the S42T mutant, we observed a large variability between different experiments. Fig. 2 shows examples of current traces and P_O_ values from two independent sets of experiments performed several months apart. The P_O_/V relationships from both sets of experiments exhibit the same voltage-dependent inactivation but the position of the V_1/2_ value is shifted by about 35 mV between the two experiments.

**Figure 3.**
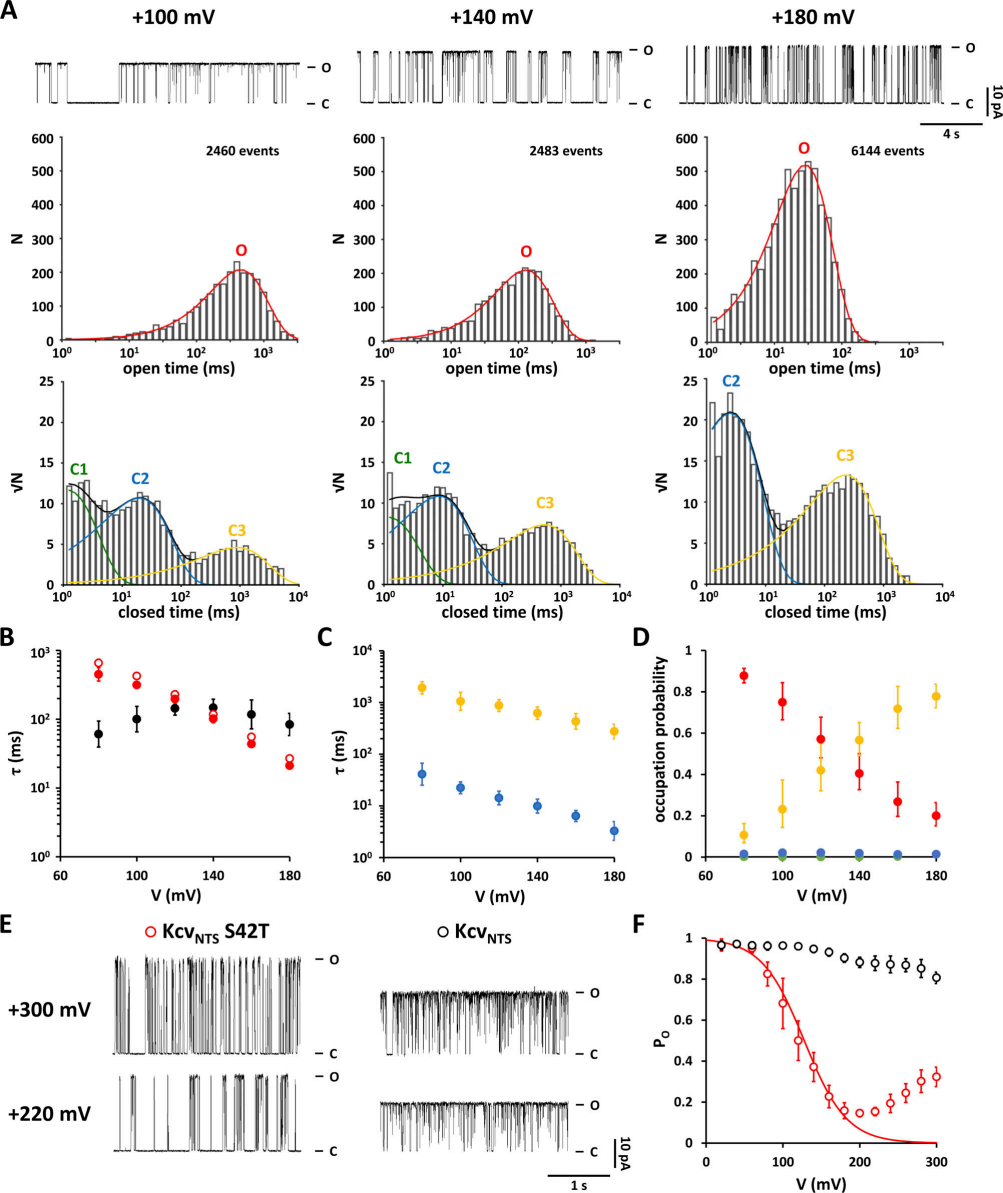
**Inactivation of Kcv**_**NTS**_ **S42T at positive potentials is caused by a voltage-dependent increase in the frequency of closings and the appearance of two voltage-dependent closed-time populations. (A)** Representative open (top) and closed (bottom) dwell-time histograms from single-channel recordings of Kcv_NTS_ S42T. Test voltages as well as a short current trace are reported above corresponding dwell-time histograms. Open dwell-time histograms show only one open dwell-time population (O) and were therefore fitted with a single exponential function (solid red line). Closed dwell-time histograms were fitted with three or two exponential functions. Closed dwell-time populations are indicated with C1 (solid green line), C2 (solid blue line) and C3 (solid yellow line). The sums of these exponentials are shown as solid black lines. The number of detected events used to construct the closed and open dwell-time histograms are indicated in the open time histograms. **(B)** Voltage-dependence of mean open (red) and mean closed (black) lifetimes calculated from dwell-time histograms as in A. **(C)** Mean lifetimes of closed dwell-time populations C2 (blue) and C3 (yellow) calculated from dwell-time histograms as in A. **(D)** Voltage dependence of occupation probabilities of open state O (red) and closed-time populations C1 (green), C2 (blue), and C3 (yellow). Data points in B–D show geometric mean ± geometric SD of three independent measurements. **(E)** Representative single-channel current traces from Kcv_NTS_ and Kcv_NTS_ S42T at high voltages of +220 and +300 mV. Closed and open levels are marked by C and O, respectively. **(F)** Open probabilities from time series like in E. Data points correspond to symbols in E and show arithmetic mean ± SD of at least three independent measurements. The solid red line shows the P_O_/V relationship expected for voltage-dependent gating based on the Boltzmann equation (Eq. 1).

To understand the mechanism of inactivation in Kcv_NTS_ S42T at positive voltages and the reason for the variability in the data, we performed a dwell-time analysis. The data show that the mean open lifetime decreases exponentially with positive voltages while the mean closed lifetime has a weak bell-shaped voltage dependency with a maximum of about +140 mV (Fig. 3 B). Close inspection of the closed dwell-time histograms for voltages between +80 and +160 mV reveals three populations C1–C3 (Fig. 3 A). C1 has a mean lifetime τ_C1_ of about 1 ms at the edge of resolution and disappears at voltages greater than or equal to +160 mV (Fig. 3 A). The frequency of events attributed to populations C2 and C3 increases with depolarization (Fig. 3 A). Calculation of the occupation probabilities reveals that the decrease in P_O_ is mainly caused by an increase in the occupation probability of C3 (P_C3_) (Fig. 3 D). Notably, the latter is not the result of an increase in the mean lifetime τ_C3_, but solely caused by an increase in closing frequency. In fact, the mean lifetimes of closed events C2 and C3 decrease almost exponentially with depolarizing voltages (Fig. 3 C). This behavior is rather unusual for a voltage-dependent gate and more reminiscent of a charged pore blocker, which can permeate through the pore as the electrical driving force increases. Therefore, we speculated that the S42T mutation may have not affected a gate in Kcv_NTS_ but increased the sensitivity of the channel to a blocker that is present in the solutions used in an undefined concentration.

To test this assumption, we performed experiments in which we clamped the WT and the mutant channel in symmetrical 100 mM KCl buffer to extreme positive voltages. This causes in Kcv_NTS_ WT only a small drop in P_O_ to about 0.8 at +300 mV (Fig. 3, E and F). The mutant channel on the other hand exhibits the expected voltage-dependent decrease in P_O_ toward zero as expected from the Boltzmann equation before rising again at voltages more positive than +200 mV (Fig. 3 F). The current traces in Fig. 3 E reveal that P_O_ rises again at extreme positive voltages because the closed times shorten to a greater extent than the closing frequency increases. This is a strong indication of a so-called punch-through behavior (French and Shoukimas, 1985; Nimigean and Miller, 2002) in which the blocking ion is relieving beyond a critical positive voltage the block by exiting through the filter to the extracellular solution rather than back toward the cytosol; this favors an increasing net conduction of K^+^ at extreme positive voltages.

### Screening of divalent cations as potential pore blockers

In search for unknown blockers in the solution, we screened the effect of divalent cations in the cytosolic solution on channel activity. These experiments were motivated by three reasons: first, the decreasing lifetimes of channel closures in the mutant is reminiscent of a Ba^2+^ block in K^+^ channels (Neyton and Miller, 1988). Second, previous data have shown that divalent cations in the cytosolic solution cause a voltage-dependent decrease in open probability in the MthK channel (Thomson et al., 2014) with a nanomolar affinity for Ba^2+^ (Guo et al., 2014). Third, high-quality KCl salts contain, according to the manufacturer’s certificates of analysis, traces of alkaline earth metal ions; the Ba^2+^, Ca^2+^, and Mg^2+^ concentrations in a 100 mM KCl solution for example can be in the range of hundreds of nanomolar.

**Figure 4.**
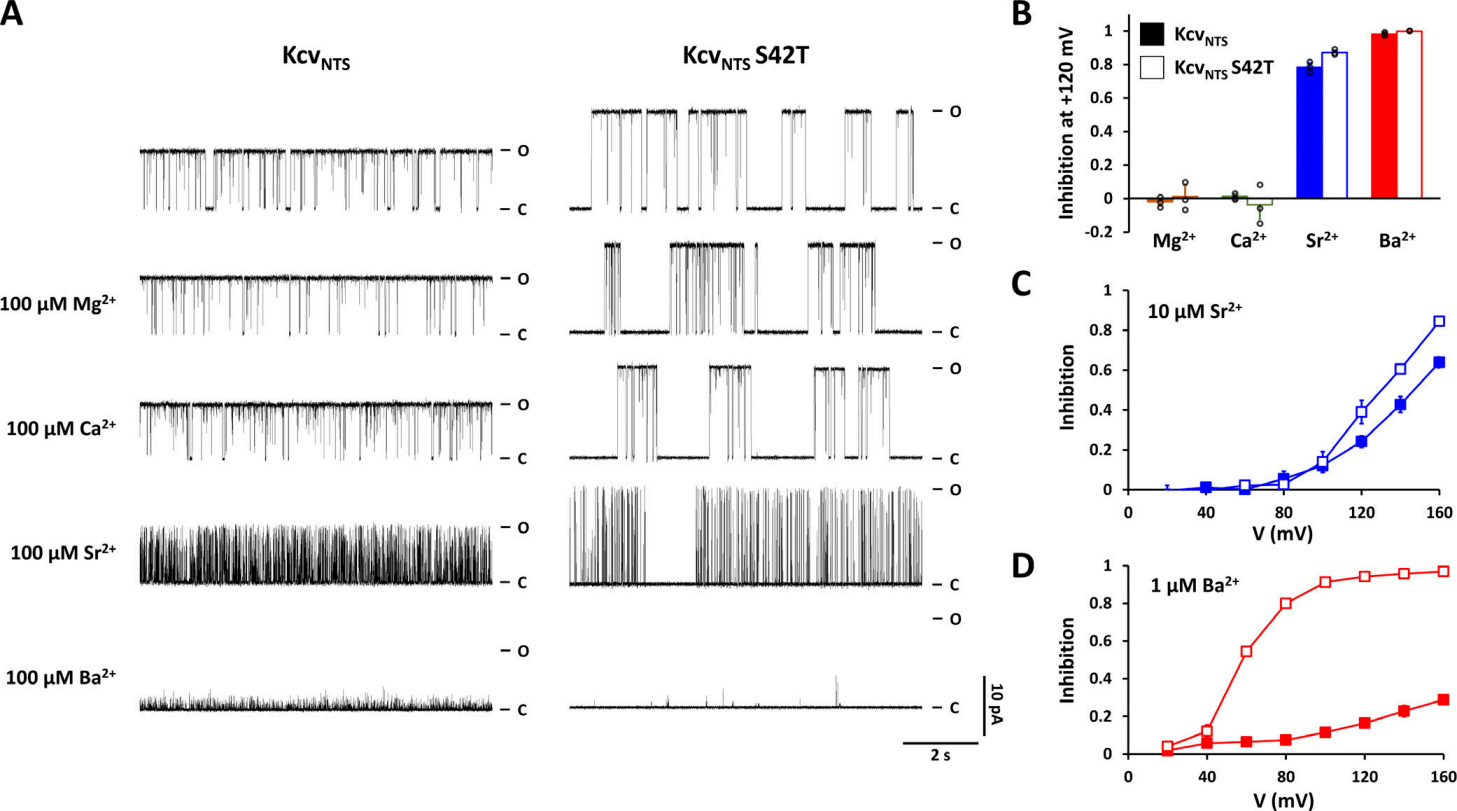
**Screening of divalent cations reveals that mutation S42T increases sensitivity to the blocking ions Sr**^**2+**^ **and Ba**^**2+**^**. (A)** Representative single-channel current traces recorded in DPhPC bilayers at +120 mV from Kcv_NTS_ and Kcv_NTS_ S42T in the absence (top row) or presence of 100 µM of the divalent cations Mg^2+^, Ca^2+^, Sr^2+^, and Ba^2+^ in the cytosolic solution. The closed and open levels are marked by C and O, respectively. **(B)** Inhibitory effect of 100 µM cytosolic Mg^2+^, Ca^2+^, Sr^2+^, and Ba^2+^ on the open probability of Kcv_NTS_ and Kcv_NTS_ S42T calculated from single channel recordings as in A. **(C and D)** Voltage-dependence of the inhibitory effect of 10 μM Sr^2+^_cyt_ (C) or 1 μM Ba^2+^_cyt_ (D) on the open probability of the Kcv_NTS_ (closed squares) and the S42T mutant (open squares). Data points in B–D show arithmetic mean ± SD of three independent measurements.

If the voltage-dependent closure of the mutant is caused by a divalent ion present as a contaminant in the solution, the mutant and the WT channel should differ significantly in their sensitivity to this ion. The data in Fig. 4 show that at a reference voltage of +120 mV only 100 μM Ba^2+^ and Sr^2+^ cause a severe block of both Kcv_NTS_ WT and Kcv_NTS_ S42T, different from the MthK channel (Thomson et al., 2014), and Ca^2+^ has at the same concentration neither an appreciable effect on the open probability of the WT nor on the mutant channel (Fig. 4, A and B). Scrutiny of the voltage-dependent inhibition by cytosolic Ba^2+^ (Ba^2+^_cyt_) and cytosolic Sr^2+^ (Sr^2+^_cyt_) at a lower concentration further reveals that the S42T mutant channel exhibits a higher sensitivity to both blocking ions compared with the WT channel (Fig. 4, C and D). This difference between the mutant and WT channels is particularly pronounced for Ba^2+^, while 1 μM Ba^2+^_cyt_ inhibits the WT channel at +160 mV by about 30%, the mutant shows inhibition of >50% already at +50 mV and of almost 100% at +160 mV (Fig. 4 D).

**Figure 5.**
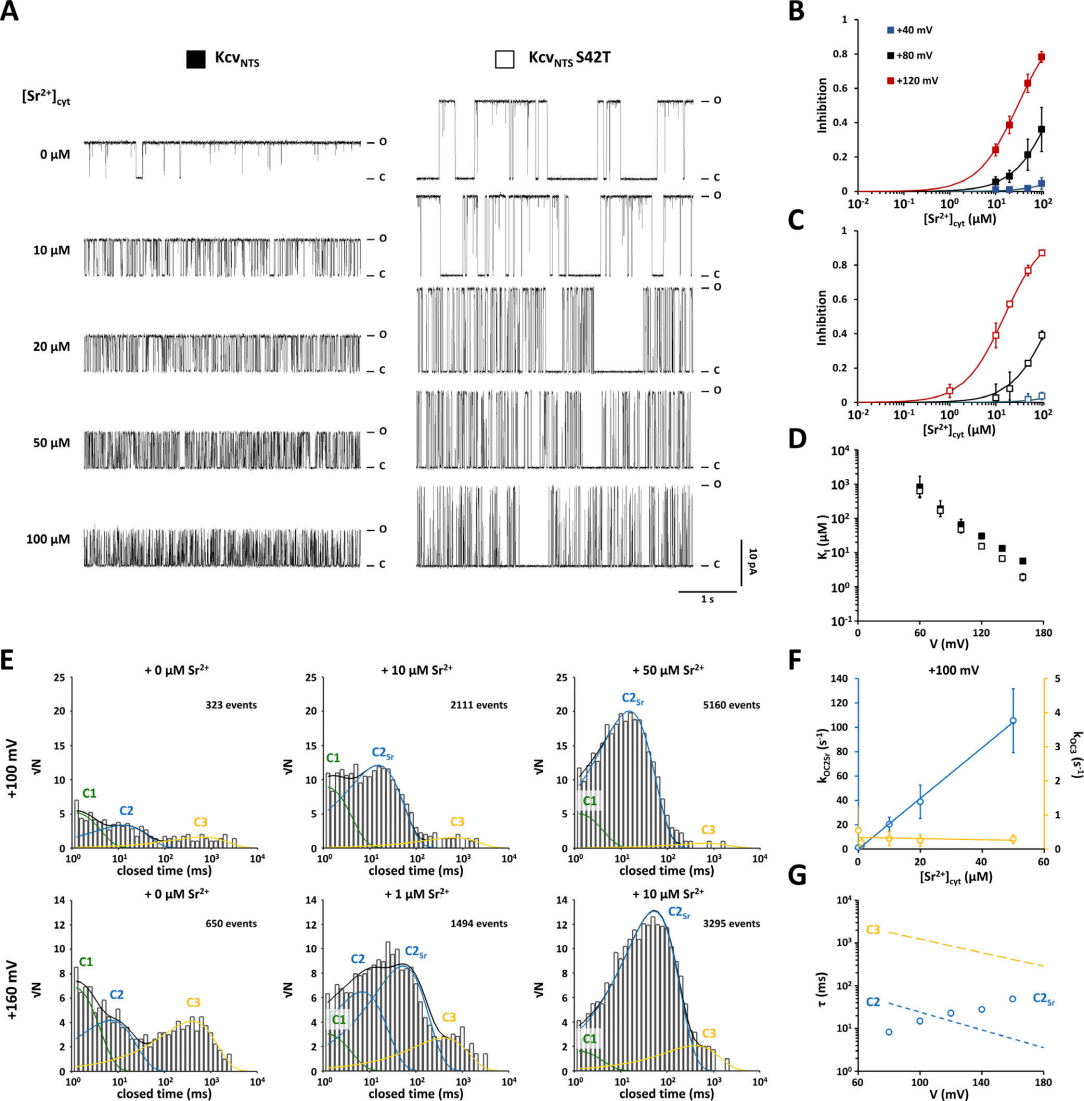
**The S42T mutation has only a minor effect on the sensitivity of Kcv**_**NTS**_ **to Sr**^**2+**^**. (A)** Representative single-channel current traces from Kcv_NTS_ and Kcv_NTS_ S42T recorded at +120 mV with increasing [Sr^2+^]_cyt_ concentrations. Closed and open levels are marked by C and O, respectively. **(B and C)** Dose–response curves for the inhibitory effect of Sr^2+^ on the open probability of Kcv_NTS_ (B) and Kcv_NTS_ S42T (C) at +40 mV (blue), +80 mV (black), and +120 mV (red). Solid lines show best fits Eq. 5. **(D)** Voltage-dependence of the inhibition constant (*K*_*I*_) of Sr^2+^ for Kcv_NTS_ (closed squares) and Kcv_NTS_ S42T (open squares) obtained by fitting Eq. 15 to dose–response curves as shown in B and C. **(E)** Representative closed dwell-time histograms from single-channel recordings of Kcv_NTS_ S42T at +100 mV (top row) and +160 mV (bottom row) in the presence of varying [Sr^2+^]_cyt_ (reported above each histogram). All histograms are from the same experiment and were constructed from 3-min single-channel traces. The number of detected events used to construct the dwell-time histograms is indicated. Histograms were fitted with up to four exponential functions. Closed dwell-time populations already present in the absence of additional Sr^2+^ are indicated with C1 (solid green line), C2 (solid blue line), and C3 (solid yellow line). The closed dwell-time population caused by Sr^2+^ is indicated by C2_Sr_ (solid blue line), respectively. Sums of all exponentials are shown as solid black lines. **(F)** [Sr^2+^]_cyt_-dependence of apparent rate constants for the transitions from the open state O to the closed states C2_Sr_ (k_OC2Sr_, blue circles) and C3 (k_OC3_, yellow circles) at +100 mV calculated from dwell-time histograms as shown in E. Solid lines show best fits to a linear function. **(G)** Voltage-dependence of mean closed lifetime of closed dwell-time population C2_Sr_. Dashed lines show exponential voltage-dependence of mean closed lifetimes of C2 and C3 from Fig. 3 C. Data points in B and E show arithmetic mean ± SD of three independent experiments. Data points in C and F show geometric mean ± geometric SD of three independent experiments. Data points in B, C, and F show arithmetic mean ± SD of three independent measurements. Data points in D and G show geometric mean ± geometric SD of three independent measurements.

**Figure 6.**
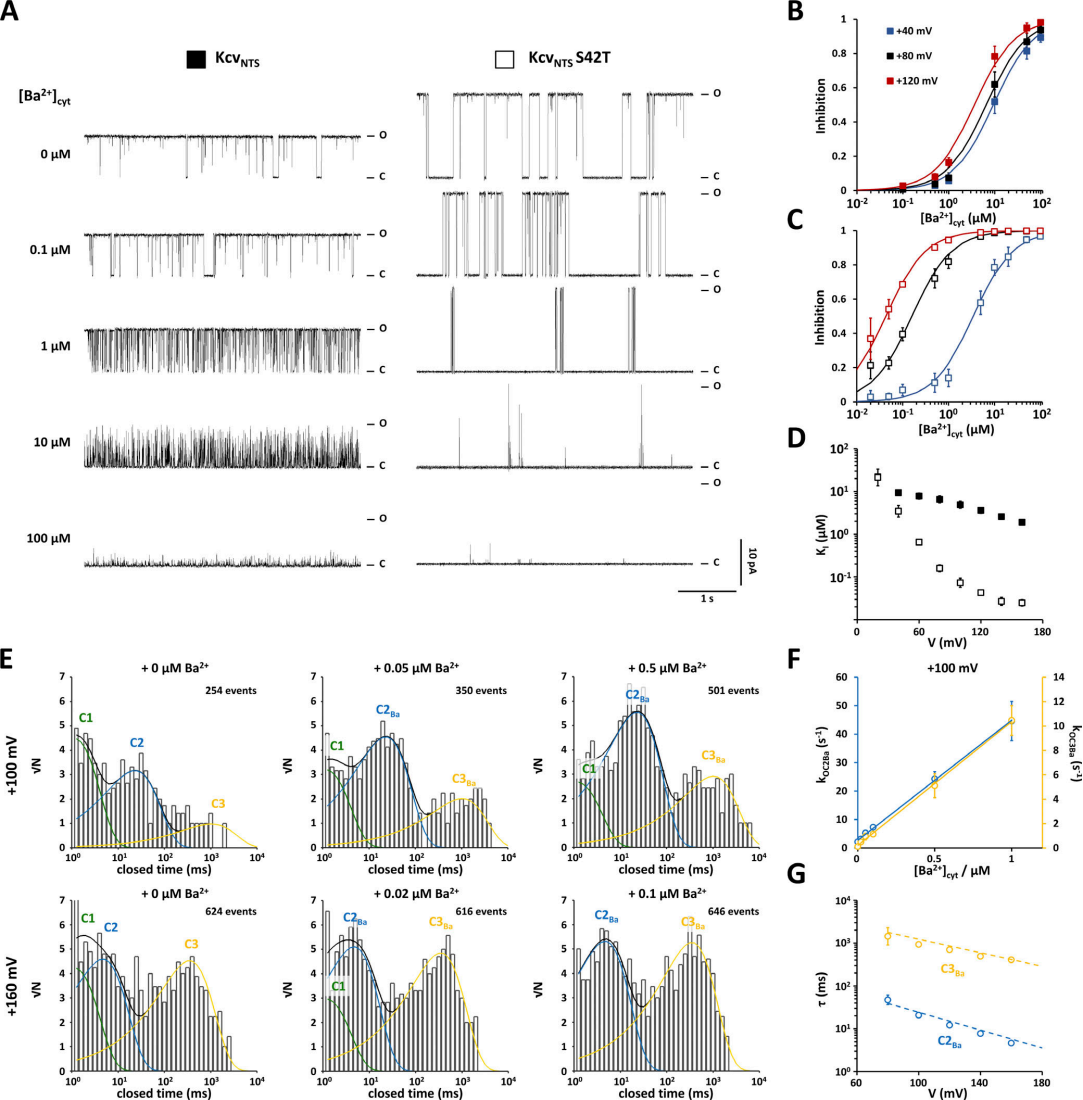
**Kcv**_**NTS**_ **S42T exhibits dramatically increased sensitivity to [Ba**^**2+**^**]**_**cyt**_**. (A)** Representative single-channel current traces from Kcv_NTS_ and Kcv_NTS_ S42T at +120 mV with increasing [Ba^2+^]_cyt_. Closed and open levels are marked by C and O, respectively. **(B and C)** Dose–response curves for the inhibitory effect of Ba^2+^ on the open probability of Kcv_NTS_ (B) and Kcv_NTS_ S42T (C) for Ba^2+^ at +40 mV (blue), +80 mV (black), and +120 mV (red). Solid lines show best fits Eq. 5. **(D)** Voltage-dependence of the inhibition constant (*K*_*I*_) of Ba^2+^ for Kcv_NTS_ (closed squares) and Kcv_NTS_ S42T (open squares) obtained by fitting Eq. 15 to dose-response curves as shown in B and C. **(E)** Representative closed dwell-time histograms from single-channel recordings of Kcv_NTS_ S42T at +100 mV (top row) and +160 mV (bottom row) in the presence of varying [Ba^2+^]_cyt_ (reported above each histogram). All histograms are from the same experiment and were constructed from 3-min single-channel traces. The number of detected events used to construct the dwell-time histograms is indicated. Histograms were fitted with up to three exponentials. Closed dwell-time populations already present in the absence of additional Ba^2+^ are indicated with C1 (solid green line), C2 (solid blue line), and C3 (solid yellow line). Closed dwell-time populations caused by Ba^2+^ are indicated by C2_Ba_ (solid blue line) and C3_Ba_ (solid yellow line), respectively. Sums of all exponentials are shown as solid black lines. **(F)** [Ba^2+^]_cyt_-dependence of apparent rate constants for the transitions from the open state O to the closed states C2_Ba_ (k_OC2Ba_, blue circles) and C3_Ba_ (k_OC3Ba_, yellow circles) at +100 mV calculated from dwell-time histograms as shown in E. Solid lines show best fits to a linear function. **(G)** Voltage-dependence of mean closed lifetimes of closed dwell-time populations C2_Ba_ and C3_Ba_. Dashed lines show exponential voltage-dependence of mean closed lifetimes of C2 and C3 from Fig. 3 C. Data points in C and F show geometric mean ± geometric SD of three independent experiments. Data points in B, C, and F show arithmetic mean ± SD of three independent measurements. Data points in D and G show geometric mean ± geometric SD of three independent measurements.

To obtain more information on the modes of inhibition, we measured dose-response curves for Sr^2+^ and Ba^2+^ induced channel block. Exemplary single-channel current traces from Kcv_NTS_ and Kcv_NTS_ S42T recorded at +120 mV with increasing [Ba^2+^]_cyt_ and [Sr^2+^]_cyt_ are shown in Fig. 5 A and Fig. 6 A, respectively. The dose-response curves can be described for all voltages with the Hill equation for a bimolecular reaction (Hill coefficient = 1):

$$Inhibtion=\frac{{\left[X^{2+}\right]}_{cyt}}{{\left[X^{2+}\right]}_{cyt}+K_{I}}\text{,}$$ (15)

with [*X*^*2+*^]_*cyt*_ being the concentration of Sr^2+^ or Ba^2+^ in the cytosolic solution and *K*_*I*_ the inhibition constant. Eq. 15 implies that the channel block is caused by binding of a single Sr^2+^ or Ba^2+^ ion in the permeation pathway.

### S42T only slightly increases the sensitivity of Kcv_NTS_ to cytosolic Sr^2+^

Sr^2+^_cyt_ induces in the WT and mutant channel short closing events (<100 ms) whose frequency increases with increasing Sr^2+^ concentration (Fig. 5 A). The *K*_*I*_ value of the Sr^2+^ block decreases for WT and S42T mutant almost exponentially from 1 mM at +60 mV by three orders of magnitude to 1 μM at +160 mV (Fig. 5 D); the *K*_*I*_ value of the mutant drops only at membrane voltages greater than +100 mV below the reference value of the WT channel. This minor difference in Sr^2+^ affinity cannot explain the decrease in P_O_ of the S42T mutant at positive voltages.

An additional argument against Sr^2+^ as blocking contaminant comes from a comparative analysis of the dwell times: the addition of Sr^2+^ to the cytosolic solution elicits only one additional population of block events (C2_Sr_, Fig. 5 E), while the decrease in P_O_ of the S42T mutant can be attributed to the voltage-dependent appearance of two closed-time populations (C2 and C3) (Fig. 3). At first glance, the mean lifetime of C2_Sr_ falls within the range of the C2 population (Fig. 5 E); at moderate positive membrane potentials, C2 and C2_Sr_ are indistinguishable (Fig. 5 E). However, at high voltages C2_Sr_ is no longer identical to C2, and the closed time histograms at +160 mV and 1 μM Sr^2+^ can only be described in a satisfying way by adding a fourth exponential function (Fig. 5 E). If we assume that each closed-time population Cj represents a separate closed state that can be reached exclusively from the open state (for details see Materials and methods and Scheme 1), we can calculate the apparent rate constants (k_OCj_) for the transitions from the open to the closed states. As expected for a concentration-dependent block, the apparent rate constant k_OC2Sr_ of the C2_Sr_ population increases linearly with [Sr^2+^]_cyt_ (Fig. 5 F). In contrast, the rate constant k_OC3_ of population C3 is unaffected by an increase in [Sr^2+^]_cyt_. However, the [Sr^2+^]_cyt_-dependent increase in the number of C2_Sr_ events causes a decrease in the amplitudes of populations C2 and C3, indicating that Sr^2+^ and the unknown contaminant cannot block the pore simultaneously. The fact that C2_Sr_ is indeed not identical with C2 becomes once again apparent from the mean lifetimes; while τ_C2_ of the closed time population C2 decreases from 41 ms at +80 mV to 6 ms at +160 mV (Fig. 3 D and dashed blue line in Fig. 5 G), the mean lifetime τ_Sr_ of Sr^2+^ block events displays the inverse voltage-dependence and increases from 5 ms at +80 mV to 50 ms at +160 mV (Fig. 5 G).

### S42T dramatically increases the sensitivity of Kcv_NTS_ to cytosolic Ba^2+^

Analysis of the Ba^2+^ block shows that the S42T mutation significantly increases the affinity for Ba^2+^_cyt_ (Fig. 6, A–D). The current traces depicted in Fig. 6 A illustrate this impressively: while 1 μM Ba^2+^ has almost no effect on the open probability of the WT channel at +120 mV, the S42T mutant is almost completely blocked at the same concentration. Furthermore, the dose-response curves reveal that the S42T mutant is for voltages greater than +60 mV sensitive to [Ba^2+^]_cyt_ far below 1 µM (Fig. 6 C). This is also reflected in the calculated *K*_*I*_ values (Fig. 6 D). For the WT channel, the *K*_*I*_ value decreases from 22 μM at +20 mV to 1.9 μM at +160 mV. In the case of the S42T mutant, the *K*_*I*_ value decreases in the same voltage range by almost four orders of magnitude from 21 µM to 25 nM. These results strengthen the suspicion that the drop in P_O_ of Kcv_NTS_ S42T at positive voltages is caused by a Ba^2+^ contamination in the test solutions used. This hypothesis is further supported by the results of the dwell-time analyses (Fig. 6, E–G). Ba^2+^_cyt_ generates two closed-time populations (C2_Ba_ and C3_Ba_), which coincide with C2 and C3 at all tested voltages (Fig. 6 E). The assumption that both populations are caused by Ba^2+^ can be demonstrated by calculating the apparent rate constants k_OC2Ba_ and k_OC3Ba_: both increase linearly with [Ba^2+^]_cyt_ (Fig. 6 F) as expected for a bimolecular reaction. Moreover, the mean lifetimes of the populations C2_Ba_ and C3_Ba_ are identical to the mean lifetimes of C2 and C3 at all voltages tested (Fig. 6 G). These results strongly suggest that the voltage-dependent decrease in P_O_ of the channel mutant Kcv_NTS_ S42T is caused by traces of Ba^2+^ in the KCl solutions used.

### Chelation of cytosolic Ba^2+^ contaminations with 18C6TA abolishes voltage-dependent closures

**Figure 7.**
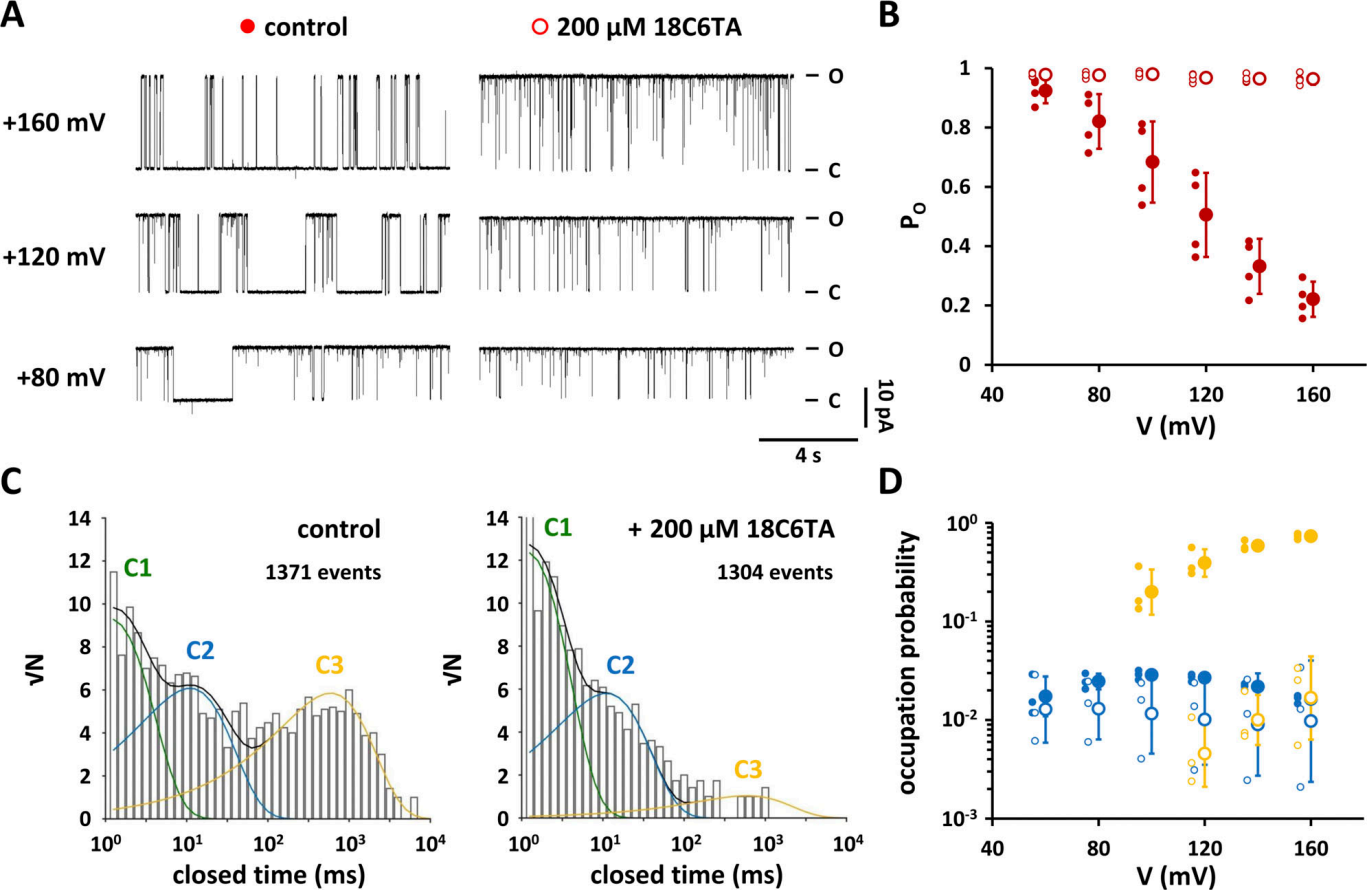
**(+)-18-Crown-6-tetracarboxylic acid (18C6TA) in the cytosolic solution eliminates long closed events in Kcv**_**NTS**_ **S42T. (A)** Representative single-channel current traces from Kcv_NTS_ S42T at indicated membrane potentials in the absence (control) and presence of 200 µM 18C6TA in the cytosolic solution. Closed and open levels are marked by C and O, respectively. **(B)** Open probabilities of Kcv_NTS_ S42T in the absence (closed red circles) and presence (open red circles) of 200 µM 18C6TA in the cytosolic solution. Data points show arithmetic mean ± SD of three independent measurements. Individual data points are shown to the left of the mean values. **(C)** Representative closed dwell-time histograms from single-channel recordings of Kcv_NTS_ S42T at +160 mV before (control) and after addition of 200 µM 18C6TA. Both histograms are from the same experiment and were constructed from 5-min single channel traces. The number of detected events used to construct the dwell-time histograms are indicated. Histograms were fitted with three exponentials indicated with C1 (solid green line), C2 (solid blue line), and C3 (solid yellow line). Sums of all exponentials are shown as solid black lines. **(D)** Occupation probabilities of closed-time populations C2 (blue) and C3 (yellow) in the absence (closed circles) and presence (open circles) of 200 µM 18C6TA in the cytosolic solution. Data points show geometric mean ± geometric SD of three independent measurements. Individual data points are shown to the left of the mean values.

The data advocate a scenario in which the S42T mutation increases the affinity of the channel for a block by Ba^2+^. The aforementioned variability in the voltage-dependent closure of the mutant (Fig. 2) may in that case only report different amounts of Ba^2+^ contaminations in the KCl salt between different lots. This predicts that a complete removal of Ba^2+^ from the buffer should eliminate the voltage-dependent closures in Kcv_NTS_ S42T. For a test of this prediction, we measured Kcv_NTS_ S42T activity before and after adding 200 µM of the macrocyclic polyether 18C6TA to the cytosolic solution. 18C6TA has a very high affinity to Ba^2+^ (K_D_ < 3.5 × 10^−10^ M, see Neyton, 1996) and was already used before for removing traces of Ba^2+^ contaminations from the buffer (Neyton, 1996; Diaz et al., 1996; Vergara et al., 1999). The single-channel traces (Fig. 7 A) and the corresponding P_O_/V relations (Fig. 7 B) show that 18C6TA almost completely abolishes long-channel closures at positive voltages. The mutant channel acquires the same high P_O_ value as the WT channel. Analysis of the closed dwell times reveals that the increase in the open probability is due to a reduction in the occupation probabilities of populations C2 and C3 (Fig. 7, C and D). C3 is even no longer detectable at voltages less than +120 mV (Fig. 7 D). The finding that C2 does not decrease to the same extent as C3 is most likely due to the fact that the S42T mutant, like the WT channel (Rauh et al., 2017), has a Ba^2+^-independent closed-time population with a similar mean lifetime to C2_Ba_. This will be discussed in detail below. The results of these experiments underscore that the voltage-dependent closures of the Kcv_NTS_ S42T mutant are not generated by a conventional gating mechanism; they are the result of a voltage-dependent block by Ba^2+^.

### Kcv_NTS_ S42T is sensitive to nanomolar concentrations of cytosolic Ba^2+^

**Figure 8.**
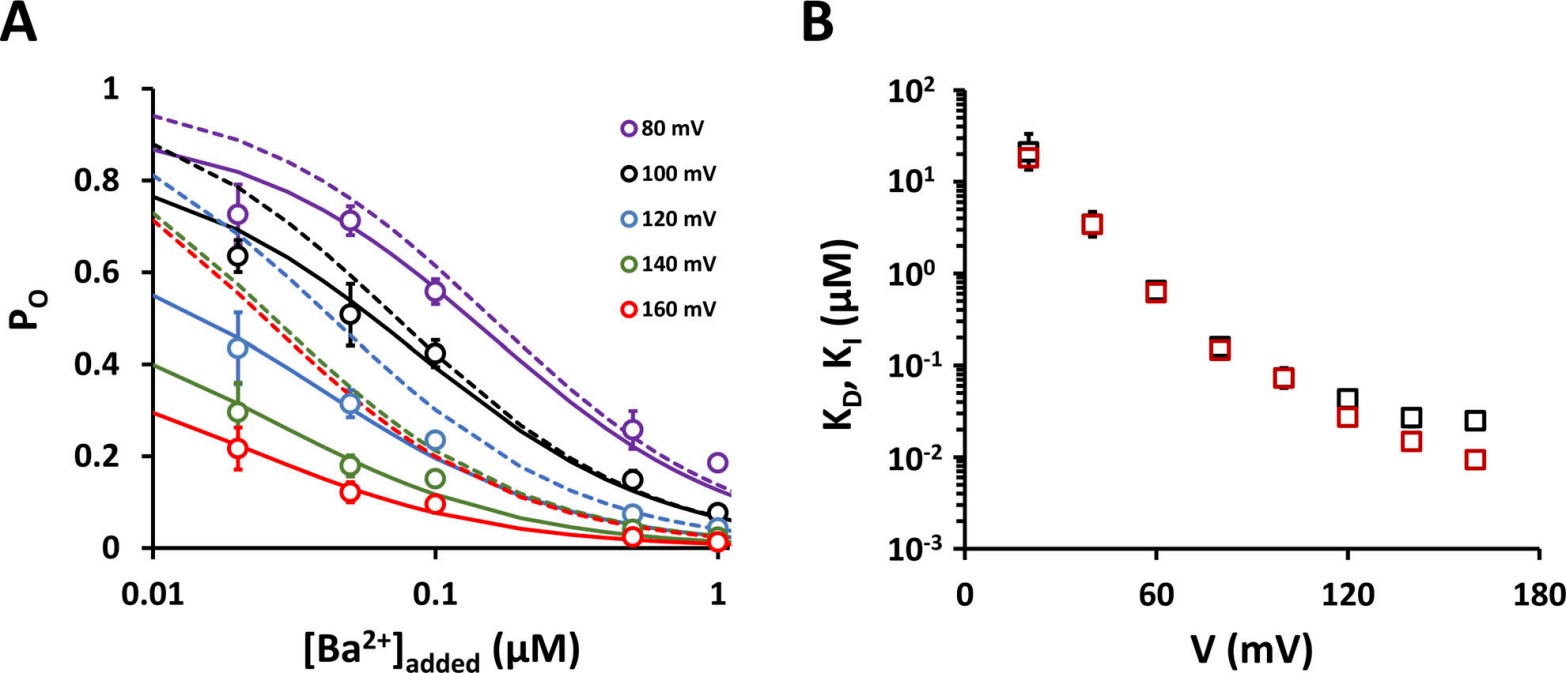
**Ba**^**2+**^ **contamination in the nanomolar concentration range accounts for a decrease in P**_**O**_ **at positive voltages in Kcv**_**NTS**_ **S42T. (A)** Open probabilities measured at constant voltages in the presence of different added Ba^2+^ concentrations. Smooth lines represent best fits with Eq. 16. Dashed lines represent the expected relationship between P_O_ and [Ba^2+^]_added_ assuming that no Ba^2+^ contamination exists and the inhibition constants (*K*_*I*_) calculated above are identical to the dissociation constants (*K*_*D*_) in Eq. 15. Data points show arithmetic mean ± SD of three independent measurements. **(B)** Comparison of inhibition constants (*K*_*I*_, black squares) from Fig. 6 and apparent dissociation constants (*K*_*D*_, red squares) obtained by fitting Eq. 16 to dose-response curves as shown in A.

The proposed voltage-dependent decrease in P_O_ by a Ba^2+^ contamination in the cytosolic solution implies that the above *K*_*I*_ values cannot be correct due to an unknown Ba^2+^ concentration in all test solutions. Hence, the affinity of the S42T mutant to Ba^2+^_cyt_ must have been underestimated at voltages greater than +60 mV. Based on the results in Fig. 7, we can assume that the open probability of the S42T mutant is close to 1 in the total absence of Ba^2+^_cyt_. Since the Ba^2+^ block can be described as a bimolecular reaction (Fig. 6), the open probability of Kcv_NTS_ S42T should obey the following equation:

$$P_{O}=\frac{1}{1+\frac{1}{K_{D}}\left({\left[Ba^{2+}\right]}_{cont.}+{\left[Ba^{2+}\right]}_{added}\right)}\text{,}$$ (16)

with [Ba^2+^]_cont._ being the unknown concentration of the Ba^2+^ contamination in the KCl solution, [Ba^2+^]_added_ the concentration of Ba^2+^ added to the cytosolic solution, and *K*_*D*_ the voltage-dependent apparent dissociation constant. The sum of [Ba^2+^]_cont._ and [Ba^2+^]_added_ represents the total [Ba^2+^]_cyt_. If we use the *K*_*I*_ values calculated above as an approximation for the *K*_*D*_ values in Eq. 16 and assume that the total [Ba^2+^]_cyt_ is equal to the added Ba^2+^ concentration, we find a significant deviation of the calculated open probabilities (Fig. 8, dashed lines) from the measured values for voltages from +80 to +160 mV; the more positive the voltage, the larger the deviation. To obtain the true *K*_*D*_ values and the concentration of the Ba^2+^ contamination, we fitted the P_O_ values as a function of [Ba^2+^]_added_ at different voltages with Eq. 16 (Fig. 8, smooth lines). The very good fits revealed that the *K*_*D*_ value for Ba^2+^ drops from 18 μM at +20 mV to about 9 nM at +160 mV. Furthermore, according to these calculations, the solutions used for channel recordings were contaminated with 12.3 ± 1.8 nM Ba^2+^. Analyzing the same solutions using inductively coupled plasma mass spectrometry (ICP-MS) gave an almost identical value of 11.9 ± 0.2 nM, confirming that channel closures of the Kcv_NTS_ S42T mutant are generated by a Ba^2+^ block. Almost the entire amount of Ba^2+^ in the KCl solutions originates from the KCl salt, as a 300-fold lower Ba^2+^ concentration could be detected in the double-distilled water that was used to dissolve the salt.

### SF properties are highly sensitive to mutations at the C-terminal end of the pore-helix

Previously, we found that not only the mutation S42T but also the mutations S42A and S42V result in the occurrence of voltage-dependent closures at positive voltages (Rauh et al., 2022). These closures can be suppressed by adding the crown ether 18C6TA to the cytosolic solution (Fig. S3), which clearly demonstrates that the voltage-dependent decrease in the open probability of the mutants S42A and S42V is also in these cases caused by traces of Ba^2+^ in the measurement solution. Interestingly, all these mutations also caused an increase in unitary conductance. To investigate the causal relationship between the two functional parameters, we substituted S42 with 13 amino acids of different sizes, hydrophobicity, and charge.

**Figure 9.**
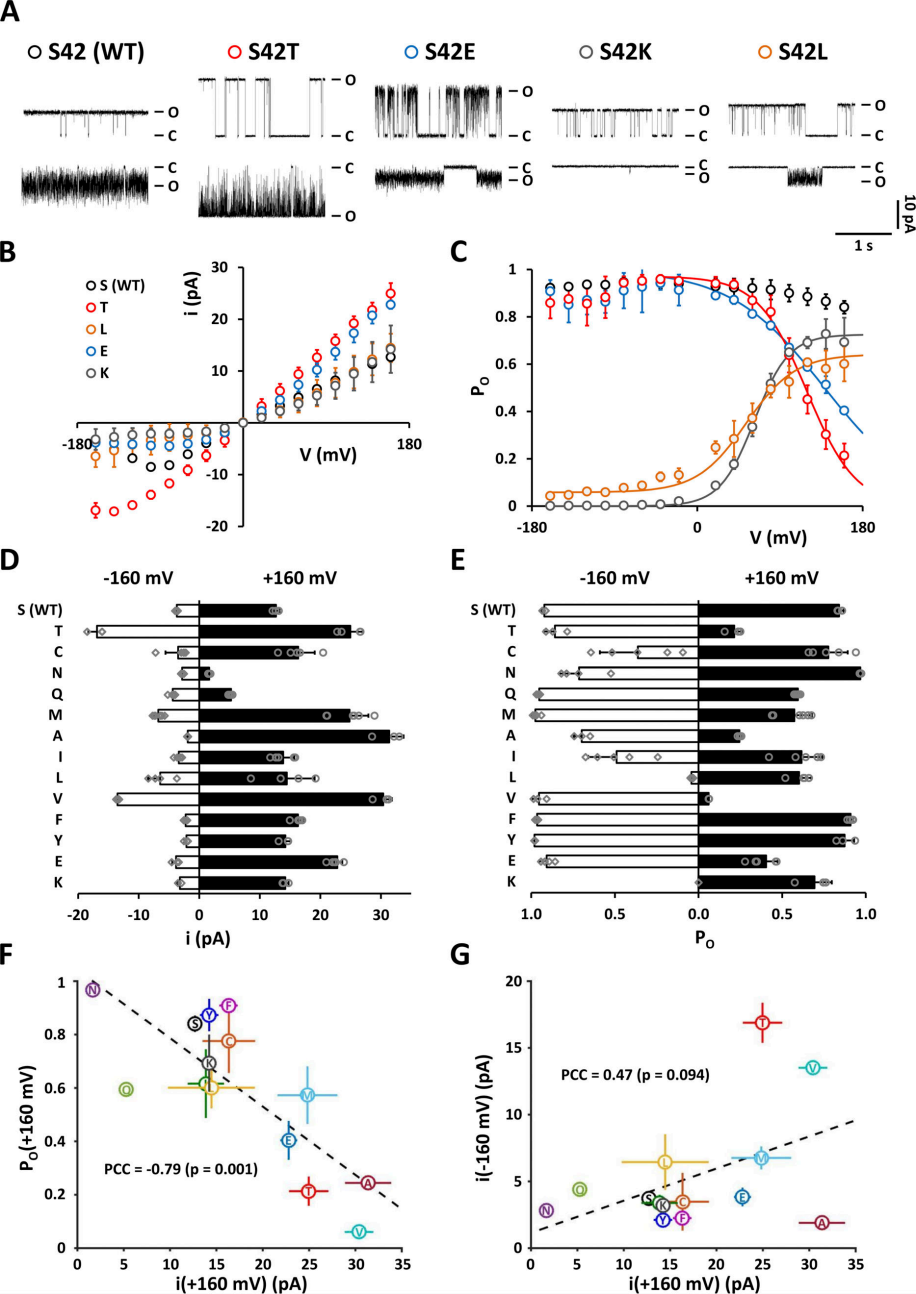
**Substitution of the critical amino acid S42 at the C-terminal end of the pore-helix has dramatic impacts on gating and K**^**+**^ **conductance of Kcv**_**NTS**_ **in an amino acid-specific manner. (A)** Representative single-channel current traces from Kcv_NTS_ (S42) and its mutants S42T, S42L, S42E, and S42K recorded in DPhPC bilayers at +120 mV (top) and −120 mV (bottom). The closed and open levels are marked by C and O, respectively. **(B and C)** Current-voltage relationships and open probabilities (P_O_) for Kcv_NTS_ variants are shown in A. Symbols as in A. **(D and E)** Open-channel current amplitudes (i) and open probabilities (P_O_) of Kcv_NTS_ (top) and various S42 mutants at −160 and +160 mV. Amino acids inserted for S42 are indicated on the left (single-letter code). **(F and G)** Scatter plots of open-channel amplitudes at +160 mV versus P_O_ at +160 mV (F) or open-channel amplitudes at −160 mV (G) for S42 mutants shown in D and E. The capital letters assigned to the data points correspond to the single-letter code of the corresponding amino acid at position 42. Dashed lines represent the best linear fit. Corresponding Pearson correlation coefficient (PCC) and *P*-value are displayed in the graph. Data points in B–G show arithmetic mean ± SD of at least three independent measurements.

Functional reconstitution of these Kcv_NTS_ mutants reveals that position 42 at the C-terminal end of the pore-helix has a key role in modulating conductance and filter gating of this channel pore: all substitutions result in functional channels and in an amino acid-specific change in unitary conductance, fast gating at negative membrane voltages, and slow gating at negative and/or positive voltages (Fig. 9). Fig. 9 A shows representative single-channel traces of four mutants in which S42 was replaced by the polar amino acid threonine (S42T), the aliphatic amino acid leucine (S42L), and the negatively and positively charged amino acids glutamate (S42E) and lysine (S42K), respectively. At positive voltages, the substitution of S42 with glutamate (E) has a similar effect on unitary conductance and P_O_ as the mutation S42T (Fig. 9, B–D). But in contrast to S42T, S42E leads to a reduction of the apparent single-channel amplitude at negative voltages, presumably due to an acceleration of fast gating kinetics (Fig. 9, A and B). Interestingly, inserting the positively charged amino acid lysine has no effect on the open-channel conductivity at positive voltages (Fig. 9, A and B) but converts Kcv_NTS_ into a strong outward rectifier (Fig. 9 C). The mutant has an open probability of virtually 0 at negative voltages, which increases with a V_1/2_ of +62.2 ± 5.5 mV and an apparent gating charge of 1.37 ± 0.16 e_0_ to a maximal Po value of about 0.7 at high positive voltages (Fig. 9 C). This outward rectification is most likely not related to the positive charge of the lysine residue since the substitution of amino acid 42 with the aliphatic amino acid leucine (L) produces a similar, albeit somewhat weaker, voltage dependence with V_1/2_ of +53.1 ± 8.4 mV and an apparent gating charge of 0.96 ± 0.33 e_0_ (Fig. 9, A and C).

The experimentally determined open-channel amplitudes and open probabilities at −160 and +160 mV of all tested channel variants are summarized in Fig. 9, D and E, respectively. It is astonishing that even (apparently) small changes in the physicochemical properties of amino acid 42 can evoke large changes in the functional properties of the channel pore (e.g., S versus T or V versus I), whereas amino acids with very different properties, such as K and L, can give rise to very similar phenotypes. Thus, it can be concluded that Kcv_NTS_ is highly sensitive to mutations at position 42. Although a simple correlation between the physicochemical properties of the amino acid at position 42 and the electrophysiological properties of the mutant cannot be identified, we find a correlation between K^+^ conductance at positive membrane voltages and the sensitivity against cytosolic Ba^2+^. When the open-channel amplitude at +160 mV is plotted against the corresponding open probability (as a measure for the sensitivity to Ba^2+^), there is a strong negative correlation with a Pearson correlation coefficient of −0.79 (P = 0.001). This means that a mutation that increases the outward conductivity of the filter for K^+^ simultaneously decreases the permeability of the filter for Ba^2+^. In contrast, the effect of mutations on the K^+^ conductance at positive voltages only weakly correlates with the apparent open-channel amplitude at negative voltages (Fig. 9 G); the latter value is an indirect measure of the stability of the filter gate at negative voltages (Rauh et al., 2022). This difference in correlation suggests that the effects of a mutation at position 42 on conductivity and gating of the K^+^ channel pore at positive and negative membrane voltages are unrelated.

### Conclusions

The miniature size Kcv channels represent, in their basic structural and functional properties, the pore module of all K^+^ channels. Among >100 known Kcv channel variants (Murry et al., 2020), Kcv_NTS_ is most useful for understanding structure/function correlates in the selectivity filter of K^+^ channels because this channel lacks a cytosolic gate and its gating is entirely dominated by structural rearrangements directly in or in the vicinity of the selectivity filter (Rauh et al., 2017). This unique combination of small size and functional reduction to a single defined gate makes Kcv_NTS_ a perfect model system for studying filter gating.

We had previously reported that mutations in the critical position 42 in Kcv_NTS_, which is equivalent to a functional hot spot for filter gating in KcsA (E71), MthK (V55), and Kv channels (V370 in Kv1.2 [Chao et al., 2010]), alter key properties of this Kcv channel. In particular, the S42T mutant shows an increase in unitary conductance as well as a stabilization of the SF gate at hyperpolarizing voltages (Rauh et al., 2022). The same mutation also generates a voltage-dependent and K^+^-sensitive closing at depolarizing voltages. This phenomenon, which was further studied here, resembles at first glance SF-mediated C-type inactivation in other K^+^ channels such as KcsA or MthK. However, the key message from the present analysis is that this voltage-dependent phenomenon in Kcv_NTS_ S42T is not generated by an intrinsic gate. Different from the MthK channel where Ca^2+^ and Sr^2+^ augment C-type inactivation (Thomson et al., 2014), channel closures in Kcv_NTS_ at positive voltages are the consequence of a hypersensitivity of the mutant to Ba^2+^_cyt_. We find that also Sr^2+^, which triggers in the MthK channel C-type inactivation (Thomson et al., 2014), causes in Kcv_NTS_ S42T voltage-dependent closure at positive voltages. But this mechanism can be excluded as an explanation for the voltage dependency of Kcv_NTS_ S42T in KCl buffer without additional divalent cations. First, the required concentrations of Sr^2+^ for this effect are much higher than the impurity of 80 ± 1 nM in a 100 mM KCl buffer as measured by ICP-MS. Also, the effect of Sr^2+^ is mechanistically different from the voltage-dependent gating of Kcv_NTS_ S42T in a KCl buffer. The data on Kcv_NTS_ S42T are hence best explained by a scenario in which nanomolar Ba^2+^ contaminations in the KCl solutions cause block events that follow a double-exponential probability density function. These block events can be significantly reduced by chelating Ba^2+^ contaminations from the solution with the Ba^2+^ selective crown ether 18C6TA. The interpretation of two distinct populations of closed times in the S42T mutant as a result of a block by Ba^2+^ traces in the KCl solutions is further supported by experiments in which the population of the very two closed dwell-time populations can be augmented by adding additional Ba^2+^ to the intracellular solution.

Such a voltage-dependent block of K^+^ channels by Ba^2+^ contaminations in the experimental solution has already been described for BK channels (Neyton, 1996; Diaz et al., 1996). Also, long-lived closed events occurring at hyperpolarizing voltages in Kir1.1 could be causally related to a Ba^2+^ block arising from contaminations in the extracellular solution (Choe et al., 2001). Considering that any experimental KCl solution contains in the absence of an appropriate chelator unavoidable traces of other ions, it must be assumed that such distinct channel closures caused by blocking events might have also been interpreted in other studies as the consequence of intrinsic gating.

The filter sequence of Kcv channels as well as their geometry is very similar to other generic K^+^ channels like KcsA or MthK (Fig. 1 A and S1 A). This implies that ion permeation as well as the mechanism of channel block by ions in the pore should be like that in other K^+^ channels. This assumption is in good agreement with experimental data showing that mutations in a position that forms the binding site for the inner filter ion lowers the affinity for the Ba^2+^ block not only in a Kir channel but also in a Kcv channel (Chatelain et al., 2009). Also, from a mechanistic point of view, the Ba^2+^ block of the Kcv_NTS_ channel shares similarities with many other K^+^ channels. Like in the case of the well-studied BK, KcsA, or MthK channels, the Ba^2+^ block of Kcv_NTS_ and its S42T mutant is very asymmetrical (Guo et al., 2014; Vergara and Latorre, 1983; Piasta et al., 2011). While an extracellular concentration of 100 μM Ba^2+^ reduces the open probability in Kcv_NTS_ and its S42T mutant by ∼50% (Rauh et al., 2022), the same concentration nearly abolishes channel activity when provided from the cytosolic side (Fig. 4).

Our data suggest a scenario in which the S42T mutation at the C-terminal end of the pore-helix augments the Ba^2+^ sensitivity of the SF in an asymmetrical manner. While the affinity to external Ba^2+^ at negative voltages remains unaffected, the sensitivity to Ba^2+^_cyt_ at positive voltages increases by several orders of magnitudes giving rise to a Ba^2+^ block at nanomolar concentrations. This impact on the structural properties of the SF must be highly selective because it does not affect the sensitivity of the channel to Mg^2+^ and Ca^2+^. While the mutation also slightly increases the sensitivity to Sr^2+^, the data underpin that the mode of blocking between the two similar divalent cations is fundamentally different.

**Figure 10.**
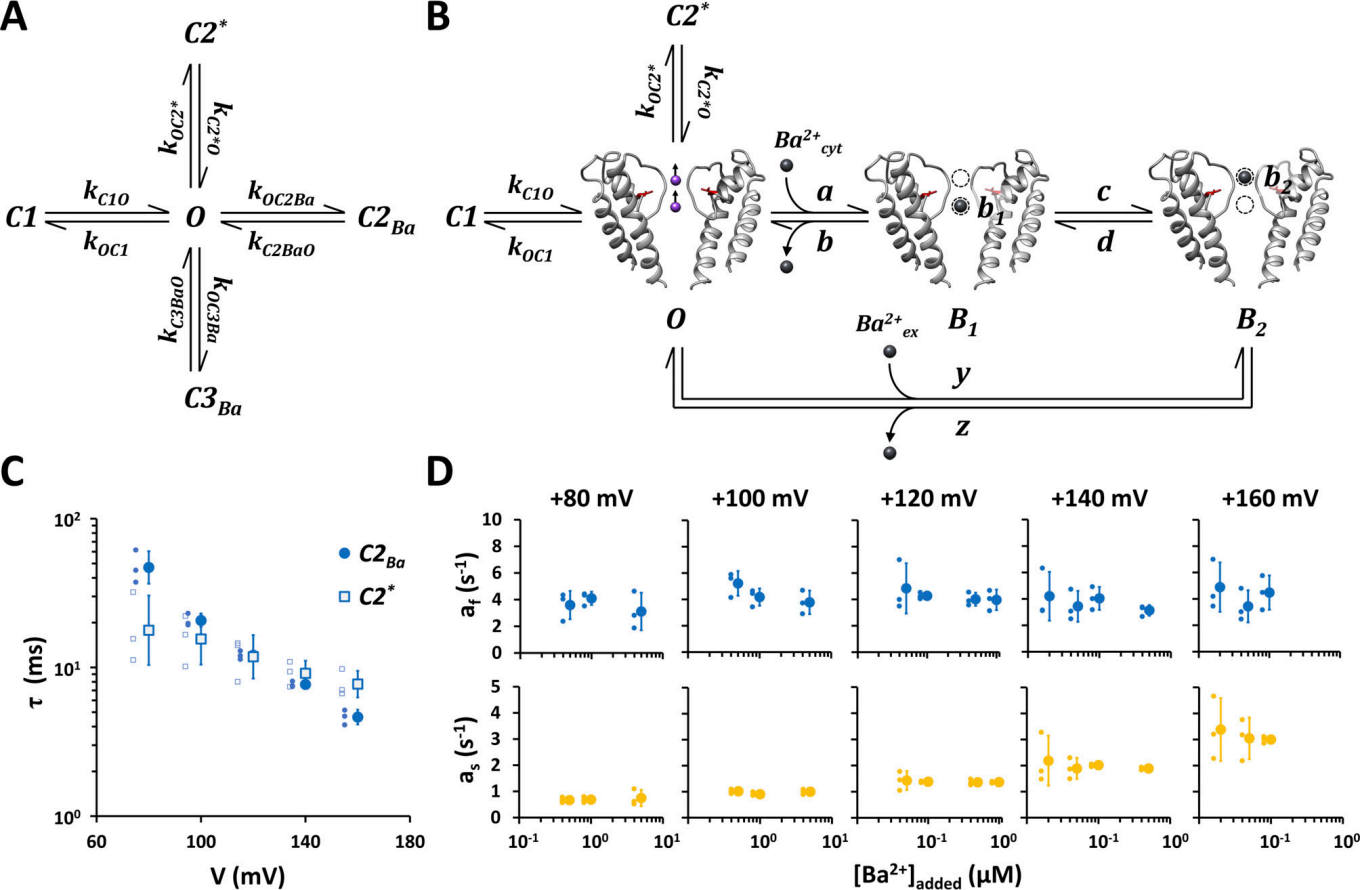
**Ba**^**2+**^ **blockade of Kcv**_**NTS**_ **S42T can be sufficiently explained by a two-state sequential blocking model. (A)** Markov-state model used to quantitatively describe open and closed events of Kcv_NTS_ S42T in the absence and presence of cytosolic Ba^2+^. This model assumes that every closed state (C) can only be reached via the open state (O); C–C transitions are not taken into account. The apparent rate constants are expressed in the form k_XY_, where X denotes the start and Y is the end state of a transition. C1 and C2* represent the closed-time populations in the complete absence of Ba^2+^_cyt_ (i.e., in the presence of 18C6TA, Fig. 7). C2_Ba_ and C3_Ba_ represent the Ba^2+^-dependent closed-time populations. **(B)** Extended Markov-state model that attributes the occurrence of the closed-time populations C2_Ba_ and C3_Ba_ to a two-state sequential blocking model. According to this model, there are two Ba^2+^ binding sites within the channel pore, of which only one binding site can be occupied by a Ba^2+^ ion at a time. A Ba^2+^ ion entering from the cytosol binds first in b_1_ driving the channel into the blocked state B_1_. This ion can then jump from b_1_ to b_2_, resulting in the blocked state B_2_. Since states B_1_ and B_2_ are non-conducting states, B_1_ and B_2_ are hidden Markov states that cannot be observed directly. Estimated positions of binding sites b_1_ and b_2_ are shown as dashed circles. Ba^2+^ ions are shown as black spheres. **(C)** Voltage-dependency of mean closed dwell-times of closed time populations C2* and C2_Ba_ determined by dwell-time analysis from single-channel recordings in the presence of 200 µM 18C6TA or after adding Ba^2+^ to the cytosolic solution, respectively. Data are geometric mean ± geometric SD of three independent measurements. Individual data points are shown to the left of the mean values. **(D)** Normalized amplitudes a_f_ and a_s_ of Ba^2+^_cyt_-dependent closed time populations C2_Ba_ and C3_Ba_, respectively, for different voltages and added cytosolic Ba^2+^ concentrations. Data points show arithmetic mean ± SD of three independent measurements. Individual data points are shown to the left of the mean values.

A detailed dose–response analysis reveals that Ba^2+^ blocking events in the S42T mutant are caused by the binding of a single Ba^2+^ ion inside the pore. This is consistent with findings for other K^+^ channels (e.g., Piasta et al., 2011; Guo et al., 2014; Choe et al., 2001). Since the dwell-time analyses revealed that Ba^2+^ generates two well-distinguishable populations of closed dwell times, it must be assumed that the SF harbors two Ba^2+^ binding sites, b_1_ and b_2_, that cannot be occupied simultaneously (Fig. 10 B). The double-exponential distribution of Ba^2+^ block times is then the result of hidden transitions of the blocking ions between these two binding sites before dissociating from the pore toward the intracellular or extracellular space. A two-state sequential Ba^2+^ blocking model has already been proposed for KcsA based on single-channel recordings (Piasta et al., 2011).

In line with this model, we assume that the closed dwell-time populations C2 and C3 of Kcv_NTS_ S42T in 100 mM KCl solution are caused by nanomolar Ba^2+^ impurities in the cytosolic solution and that these block events can be explained by a two-state sequential blocking model. This predicts that the reduction of the free cytosolic Ba^2+^ concentration by the crown ether 18C6TA abolishes these components in the closed dwell-time histograms. This effect was indeed observed for C3, whereas the C2 component was only slightly reduced. This result can be explained by the existence of an intrinsic closed state C2* (Fig. 10 A), which happens to have a similar mean lifetime as the Ba^2+^_cyt_-induced block-time population C2_Ba_. Dwell-time analyses of the single-channel traces recorded in the presence of 200 µM cytosolic 18C6TA support this assumption: in contrast to the mean lifetime of C2_Ba_, the mean lifetime of C2* is almost voltage-independent (Fig. 10 C). The observation that the mean lifetimes of the Ba^2+^-induced closed-time population C2_Ba_ coincide for all tested voltages with the mean lifetimes of C2 (Fig. 6 G) further suggests that the C2 events occurring in standard KCl solution are essentially caused by Ba^2+^ block events masking the activity of the intrinsic gate.

To further test the accuracy of the two-state blocking model, we analyzed the Ba^2+^ dependency of C2_Ba_ and C3_Ba_. Since the transitions between the blocked states B_1_ and B_2_ (rate constants c and d in Fig. 10 B) and the dissociation into the intracellular and extracellular space (rate constants b and z in Fig. 10 B) are according to the model [Ba^2+^]-independent, the renormalized amplitudes a_f_ of the fast component C2_Ba_ and a_s_ of the slow component C3_Ba_ should be [Ba^2+^]-independent. The renormalized amplitudes a_f_ and a_s_ can be calculated from Eqs. 3a and 3b given in Piasta et al. (2011) from the rate constants b, c, d, and z of the blocking model (Fig. 10 B) and the mean lifetimes τ_f_ (τ_C2Ba_) and τ_s_ (τ_C3Ba_) of the fast and slow components, respectively. Renormalization (i.e., mathematical removal of the fast component C1) was done according to Eqs. 14a and 14b.

To test this model prediction we calculated a_f_ and a_s_ from dwell-time data as shown in Fig. 6 for different voltages and cytosolic Ba^2+^ concentrations. The results shown in Fig. 10 D indeed confirm the model prediction in that the renormalized amplitudes are independent of the Ba^2+^ concentration. A Ba^2+^ dependency would have been expected if C2_Ba_ and C3_Ba_ were caused by two independent processes.

The equivalent model to that in Fig. 10 B for the Ba^2+^ block of KcsA outward current (Piasta et al., 2011) suggests that the S42T mutation may have increased in Kcv_NTS_ the Ba^2+^ affinity of the SF by creating a deeper energy well for Ba^2+^ at one of these two binding sites. This model predicts that the same mutation should also augment the sensitivity for the Ba^2+^ block from the extracellular solution. However, since previous experiments had already shown that the S42T mutant does not increase the sensitivity of the channel to external Ba^2+^ (Rauh et al., 2022), this model can be refused. On the contrary, the S42T mutation decreases the binding rate for extracellular Ba^2+^ at moderate negative voltages (Rauh et al., 2022). This supports an alternative model in which the increased asymmetrical Ba^2+^ block is explained by an elevated energy barrier for the release from the outermost binding site into the external solution (rate constant z in Fig. 10 B). In the context of the debate on whether K^+^ channels—including Kcv_NTS_—have one or two binding sites, the experimental data can also be explained by a single Ba^2+^ binding site in which the energy barrier for blocker release fluctuates between two states with a low and high barrier. The S42T mutation may in this case augment the propensity for the high energy barrier state. The question of a suitable blocking model and the structural alterations underlying the hypersensitivity of Kcv_NTS_ S42T to Ba^2+^_cyt_ at positive voltages will be addressed in a separate study.

All K^+^ channels have the same overall architecture in which the selectivity filter is anchored to the pore helix. A critical position in this arrangement is E71 in KcsA and the respective amino acids are in the equivalent position in other channels. This can be a valine in Shaker and in MthK, but also a serine in Kcv channels. Our data and those from others impressively show that any kind of mutation, even the very conservative exchange of Ser for Thr, has profound impacts on functional properties of the channel in which the mutation was made. While the original data on channel gating with the E71A mutation in KcsA were interpreted in the context of an essential hydrogen bond, which anchors the filter to the pore helix, it was already obvious from other mutations in this work, that the structure/function correlates must be much more complex and include other types of interactions. The high degree of sensitivity of this site to mutations and the functional diversity with respect to unitary conductance, gating, and blocker sensitivity, which can be generated by single amino acid substitutions in different K^+^ channels (e.g., for Kcv, Fig. 9 in this study or KscA, Fig. 2 in Chakrapani et al. [2011]), suggest that this site creates together with its interaction partners a central modulator for a fine-tuning of selectivity filter properties. The amino acid diversity of this site in different types of K^+^ channels (Prole and Marrion, 2012) presumably determines the immense functional diversity of different native K^+^ channels. Because of this sensitive anchoring of the non-flexible filter domain to the pore helix, any small conformational change in this area can result in dramatic effects on the filter and filter-related properties such as conductance, gating, and voltage dependency.

The present data also offer conceptual insights into the mechanism of filter gating in that they advocate a transient channel block as a form of gating. Our results suggest a scenario in which minute alterations in the filter structure, which are here introduced by a mutation in the critical connection between the pore helix and filter, can result in extended dwell times of Ba^2+^ in filter binding sites. This voltage-dependent filter block appears then on the functional level as a form of channel gating. Based on data with the ROMK2 channel it is reasonable to speculate that this type of filter block is not restricted to Ba^2+^; also, the permeant K^+^ ion itself may remain at certain filter configurations for longer times in its binding sites giving rise to distinct gating events (Choe et al, 1998). In fact, the short closures of the Kcv_NTS_ S42T channel at a positive voltage in the presence of crown ether (Fig. 7 A) might reflect such K^+^ block-induced gating. The fact that the equivalent position S42 in Kcv_NTS_ has also in other K^+^ channels critical importance for filter gating lets us speculate, that this kind of gating may not only be introduced by mutations. Also, external cues, which modulate, like in K2P channels, filter gating, may in this way generate subtle changes in the filter with the effect of introducing conformations in which K^+^ or other ions that can penetrate the SF bind and transiently block the ion flow.

## Data Availability

The data are directly available from the corresponding author upon reasonable request by email.
